# Generating Planar Trajectories for Neptunian System Exploration Using Motion Primitives

**DOI:** 10.1007/s40295-025-00545-z

**Published:** 2026-02-10

**Authors:** Giuliana E. Miceli, Natasha Bosanac

**Affiliations:** Colorado Center for Astrodynamics Research, Smead Department of Aerospace Engineering Sciences, University of Colorado Boulder, Boulder, CO 80303 USA

**Keywords:** Motion primitives, Spacecraft trajectory design, Multi-body gravitational systems, Neptunian system

## Abstract

This paper uses a motion primitive approach to automatically generate constrained spacecraft trajectories for Neptunian system exploration. Motion primitives are generated as smaller building blocks of motion that summarize periodic orbits and arcs along stable and unstable manifolds of selected orbits in the Neptune-Triton circular restricted three-body problem. The sequential composability of these motion primitives is represented by a graph that also incorporates path and maneuver constraints. This graph is searched using a k-best paths algorithm to generate multiple motion primitive sequences. These sequences are transformed into an array of geometrically diverse initial guesses. After corrections and optimization, the resulting tradespace of continuous, constrained trajectories with impulsive maneuvers is analyzed. This approach is applied to two planar trajectory design scenarios in the Neptunian system: high-energy insertion into a Neptune-centered science orbit after interplanetary arrival and low-energy transfers between science orbits centered around each of Neptune and Triton.

**Supplementary Information:**

The online version contains supplementary material available at 10.1007/s40295-025-00545-z.

## Introduction

The only two planets within our solar system that have not been visited for substantial periods of time are Uranus and Neptune [1]. Often labeled as ice giants, these two planets are predominantly composed of ice-forming elements, e.g., oxygen and carbon [1]. Furthermore, Uranus and Neptune are significantly more massive than terrestrial planets and possess many moons [1]. There are still many unknowns about the nature of the ice giants, such as their formation, bulk, and atmospheric composition, and the presence of water oceans on their moons [1]. These outstanding questions can benefit from a dedicated planetary exploration mission. Inspired by studies and proposals on such missions, this paper focuses on designing spacecraft trajectories within the Neptunian system.

Several researchers have designed spacecraft trajectories within various dynamical models of the Neptunian system. One common approach involves using patched conics to approximate the gravitational environment of a multi-body system as a sequence of two-body problems [2]. This approach has been employed in mission concept studies presented by Marley et al. [3] and Masters et al. [4], who used multiple successive flybys to study Neptune’s main moon, Triton. More complex approaches include modeling the point mass gravitational influence of the planet and a single moon on the spacecraft using the circular restricted three-body problem (CR3BP). This dynamical model has been used by Melman et al. to compute a transfer from a Neptune-centered orbit to a circular near-polar orbit around Triton in the Neptune-Triton CR3BP [5].

A common approach to spacecraft trajectory design in a multi-body system leverages dynamical systems techniques to explore the solution space and then construct an initial guess [6]. In an approximate dynamical model such as the CR3BP, equilibrium points, periodic orbits, quasi-periodic orbits, mean motion resonances, and hyperbolic invariant manifolds are often computed. These fundamental solutions and more general trajectories have been analyzed graphically using a variety of techniques such as Poincaré maps [6], Tisserand–Poincaré graphs [7], flyby maps [8], symplectic twist maps [9], and fast Lyapunov indicator maps [10]; and used to construct a database [11, 12]. Arcs sampled from distinct solutions are then assembled in a sequence, along with maneuvers, to form an initial guess for a complex trajectory. An initial guess is then corrected in a higher-fidelity model, and optimization may be applied to lower the total maneuver magnitude. However, with the existing approaches, initial guess construction and design space exploration can become challenging and time-consuming tasks due to the diversity of the continuous solution space in a chaotic system; the presence of hardware, mission, and operational constraints; and heavy reliance on a trajectory designer for low-level and high-level decision-making. Inspired by these challenges, researchers in the astrodynamics community have worked to automate the trajectory design process in the CR3BP. These efforts have included, but are not limited to, using graph-based searches of segments along families of periodic orbits [13, 14], reinforcement learning [15–17], and sampling-based kinodynamic planning [18].

Similar challenges have emerged across a wider variety of path planning problems. To address these challenges, motion primitives have been defined throughout the literature as building blocks of motion that support the construction of complex paths within an environment [19]. These motion primitives have summarized groups of paths with a similar geometry and are typically extracted via manual labeling, clustering, or basis function approximations [20–23]. These motion primitives have been used to reformulate a continuous-time path planning problem as a discrete graph search problem. For instance, Frazzoli et al. [20] as well as Majumdar and Tedrake [24] have constructed motion primitive graphs by defining the nodes as motion primitives and using directed edges between the nodes of sequentially composable primitives. Graph search algorithms are then used to extract sequences of composable motion primitives to form a complex trajectory. This approach has been successfully used in a variety of problems, including path planning for autonomous vehicles [20, 24].

Smith and Bosanac previously introduced using a motion primitive approach to design spacecraft trajectories in a multi-body system [25]. First, they used clustering to extract motion primitives summarizing periodic orbits and their hyperbolic invariant manifolds in the CR3BP [26]. Then, the continuous-time trajectory design problem is reformulated to a discrete graph search problem to generate sequences of motion primitives [25]. An initial guess was constructed from each primitive sequence and corrected via collocation and multi-objective optimization to generate a continuous path in the CR3BP [25]. This approach was then used to design spacecraft transfers with impulsive maneuvers between selected libration point orbits in the Earth-Moon CR3BP [25]. This application demonstrated the automatic generation of geometrically distinct trajectories that exist across a broader tradespace.

This paper presents an updated motion primitive approach to trajectory design. First, a new procedure, presented by Gillespie, Miceli, and Bosanac [27], is used to generate primitives from groups of geometrically similar arcs. This new approach improves the quality and accuracy of the groups of geometrically similar arcs used to extract motion primitives, supports generating primitives to summarize arcs that visit various regions of a system, and leverages a single set of prespecified governing parameters. Then, this paper presents a new formulation of a motion primitive-informed graph that summarizes the sequential composability of pairs of primitives. Compared to prior work, this new formulation updates the sequential composability condition, connects only composable primitives, reflects traversing segments of each primitive, and incorporates path constraints. A new graph search procedure is then used to generate multiple, unique primitive sequences. Building upon prior work, this new approach produces composable primitive sequences that satisfy path constraints, is computationally less intensive, and is capable of producing longer and more diverse primitive sequences. Finally, the procedure used to transform a primitive sequence to an initial guess that predicts a nearby, continuous trajectory is reformulated. This new approach constructs each initial guess as the combination of arcs resembling each primitive in the sequence that globally minimizes a function of the state discontinuity.

This paper demonstrates the updated motion primitive approach by designing planar transfers within the Neptunian system. Each step of the technical approach is first demonstrated through a foundational trajectory design problem: designing an array of geometrically distinct planar transfers from an $L_1$ Lyapunov orbit to an $L_2$ Lyapunov orbit in the Neptune-Triton CR3BP. Then, the updated motion primitive approach is applied to two scenarios in the Neptune-Triton CR3BP: 1) high-energy insertion into a Neptune-centered science orbit following interplanetary arrival, and 2) low-energy transfers between science orbits centered around each of Neptune and Triton. Selected transfers in each case are also corrected in an ephemeris model. These examples demonstrate that the motion primitive approach automatically generates a geometrically diverse set of trajectories that resemble their initial guesses, thereby supporting analysis of the associated tradespaces.

## Dynamical Models

This paper uses two dynamical models to describe the motion of a spacecraft in the Neptunian system. The low-fidelity Neptune-Triton CR3BP is used to generate fundamental solutions such as libration points, periodic orbits, and hyperbolic invariant manifolds to support the efficient computation of time-independent motion primitives, initial guesses, and optimal transfers. Then, selected trajectories are corrected in a higher-fidelity point mass ephemeris model of Neptune and its inner moons.

### Circular Restricted Three-Body Problem

In the CR3BP, the primary bodies are Neptune and Triton, whereas the third body is the spacecraft. This spacecraft is assumed to possess a negligibly small mass compared with the primaries. Because the masses of Neptune and Triton are approximately $1.02436 \times 10^{26}$ kg and $2.1408 \times 10^{22}$ kg [28], this assumption is reasonable. Neptune and Triton are modeled with constant, point mass gravitational fields while following circular orbits about their barycenter [29]. Because the mean eccentricity of Triton’s orbit around Neptune is 0.000016 [30], this final assumption is reasonable.

Trajectories in the CR3BP are generated in a rotating frame using nondimensional quantities. In the Neptune-Triton rotating frame, the origin is located at the system barycenter and the axes $\hat{\boldsymbol{x}},\hat{\boldsymbol{y}},\hat{\boldsymbol{z}}$ are defined as follows: $\hat{\boldsymbol{x}}$ points from Neptune to Triton, $\hat{\boldsymbol{z}}$ is aligned with the orbital angular momentum vector of the primary bodies, and $\hat{\boldsymbol{y}}$ completes the orthogonal, right-handed triad [29]. Mass quantities are normalized using $m^* \approx 1.02457 \times 10^{26}$ kg, the sum of the masses of the primaries [30]. Length quantities are normalized using $l^*=354,760$ km, the average distance between the primaries [30]. Finally, the characteristic quantity for time is calculated as $t^* \approx 8.081353 \times 10^{4}$ s to set the mean motion of the primaries equal to unity [29].

The nondimensional equations of motion governing a spacecraft in the CR3BP are expressed in the Neptune-Triton rotating frame. The state of the spacecraft is defined as $\boldsymbol{x} = [x,y,z,\dot{x}, \dot{y}, \dot{z}]^T$, and the dot notation indicates a time derivative with respect to an observer fixed in the rotating frame. Then, the equations of motion are written in the rotating frame as [29]

1 $$\begin{aligned} \ddot{x} = 2\dot{y} + \frac{\partial U^*}{\partial x}\text {,}\quad \ddot{y} = -2\dot{x} + \frac{\partial U^*}{\partial y}\text {,}\quad \ddot{z} = \frac{\partial U^*}{\partial z} \end{aligned}$$

where

$$\begin{aligned} U^*= &   \frac{1}{2}(x^2 + y^2) + \frac{(1 - \mu )}{r_1} + \frac{\mu }{r_2}\\ r_1= &   \sqrt{(x+\mu )^2 + y^2 + z^2} \quad \text {and} \quad r_2 = \sqrt{(x-1+\mu )^2 + y^2 + z^2} \end{aligned}$$

In these expressions, the mass ratio of the Neptune-Triton system is $\mu \approx 0.00020895$. This model possesses only one integral of motion, equal to $C_J = 2U^* - \dot{x}^2 - \dot{y}^2 - \dot{z}^2$ and labeled the Jacobi constant [29].

### Ephemeris Model

An ephemeris model is constructed to capture the point mass gravitational influences of Neptune, Triton, and six inner moons on the spacecraft. In this model, each body is assumed to possess a constant mass, whereas the spacecraft is assumed to possess a negligible mass in comparison. Furthermore, each celestial body is modeled to travel along paths described by the ephemerides in the following kernels provided by NASA’s Navigation and Ancillary Information Facility [31, 32]: DE440 [33], nep097, and nep095. Although there are sixteen known moons of Neptune, these ephemerides only contain accurate gravitational parameters for the six inner moons Naiad, Thalassa, Despina, Galatea, Larissa, and Proteus, and Neptune’s major moon, Triton [28]. Additionally, the naif0012 file supplies leap second information for time calculations.

The equations of motion for the spacecraft in the point mass ephemeris model are formulated relative to Neptune and in an inertial frame. In this paper, this inertial frame is defined with its origin at the center of Neptune and the axes $\hat{\boldsymbol{X}},\hat{\boldsymbol{Y}},\hat{\boldsymbol{Z}}$ of the International Celestial Reference Frame [34]. The dimensional state of the spacecraft relative to Neptune in this inertial frame is defined as $\boldsymbol{X}_{N,sc} =[X,Y,Z,X',Y',Z']^T=[\boldsymbol{R}_{N,sc},\boldsymbol{V}_{N,sc}]^{T}$ where the subscripts *N* and *sc* indicate Neptune and the spacecraft, respectively, and the notation $(.)'$ indicates a time derivative with respect to an observer fixed in the inertial frame. With these definitions, the following equations of motion govern the path of the spacecraft in the ephemeris model [2]:

2 $$\begin{aligned} {{\boldsymbol{R}}}^{\prime \prime }_{N,sc} = -GM_{N}\left( \frac{{\boldsymbol{R}}_{N,sc}}{R^{3}_{N,sc}}\right) + G \sum ^{ N_b}_{i=1} M_{i}\left( \frac{{\boldsymbol{R}}_{sc,i}}{R^{3}_{sc,i}} - \frac{{\boldsymbol{R}}_{N,i}}{R^{3}_{N,i}} \right) \end{aligned}$$

where $M_i$ is the mass of body *i*, *G* is the universal gravitational constant, $\boldsymbol{R}_{i,j}$ is the position vector measured from body *i* to body *j*, and $N_b=7$ is the selected number of Neptunian moons.

## Technical Approach

This paper leverages and improves upon prior work by Smith and Bosanac that introduced a motion primitive approach to spacecraft trajectory design in a multi-body system [25]. This section presents relevant background and a detailed description of each of the five steps of the updated technical approach. Each step is demonstrated using a foundational trajectory design problem: designing a planar transfer from an $L_1$ Lyapunov orbit to an $L_2$ Lyapunov orbit at nearby values of the Jacobi constant in the Neptune-Triton CR3BP. Flowcharts and tables summarizing the process and governing parameters are provided as supplementary material (Online Resource 1).

### Step 1: Generating Motion Primitives

Motion primitives are generated in the planar Neptune-Triton CR3BP to supply the building blocks of complex spacecraft trajectories. Similar to their use by Smith and Bosanac [26], motion primitives are extracted from selected families of periodic orbits and arcs along hyperbolic invariant manifolds. However, this paper uses an updated procedure presented by Gillespie, Miceli, and Bosanac [27] to compute a library of motion primitives that discretely summarize a segment of the continuous phase space.

#### Computing Periodic Orbits and Stable or Unstable Manifolds

Periodic orbits are periodic in the rotating frame after a minimal time labeled the period [29]. This paper uses the following families of planar periodic orbits in the CR3BP: $L_1$, $L_2$, and $L_3$ Lyapunov orbits; Triton-centered distant retrograde orbits (DROs), distant prograde orbits (DPOs), and low prograde orbits; and families associated with various mean motion resonances. In the CR3BP, a *p*:*q* resonant orbit completes *p* revolutions around Neptune in the time that Triton completes approximately *q* revolutions in the inertial frame [35, 36]. Because multiple *p*:*q* resonant orbits exist, each orbit is labeled as $\pm x \pm h$: the sign of the first term indicates whether the periapsis or apoapsis defining the initial state lies in the $+\hat{\boldsymbol{x}}$ or $-\hat{\boldsymbol{x}}$ direction relative to Neptune, whereas the sign of the second term indicates whether the orbital angular momentum vector is aligned with the third axis of a general inertial reference frame centered on Neptune that is also aligned with $\hat{\boldsymbol{z}}$.

Members of each periodic orbit family are numerically generated using multiple-shooting and continuation. First, an initial guess for one member is generated via a stability analysis of the nearby equilibrium point [29] or continuation from a lower-fidelity model [35–37]. Each initial guess is then discretized into several arcs with an equal integration time. The initial, nondimensional state along those arcs in the rotating frame and the integration time form the free variable vector. A constraint vector is then defined to enforce continuity between neighboring arcs and periodicity. The free variables are iteratively updated via Newton’s method until the norm of the constraint vector equals zero to within a tolerance of $10^{-10}$. Once a single periodic orbit has been generated, a nearby orbit along the family is computed via pseudo-arclength continuation. This process is repeated until a member of the family intersects one of the primaries, corrections fail, or a specified number of members have been computed.

The stability of a periodic orbit is computed from the eigenvalues of its monodromy matrix, i.e., the state transition matrix computed along one period of the orbit [29]. Two non-trivial and reciprocal pairs of eigenvalues indicate the qualitative behavior of motion near the periodic orbit. If a pair of non-trivial eigenvalues lies on the unit circle in the complex plane, bounded trajectories exist in the vicinity of the periodic orbit. Otherwise, the periodic orbit possesses stable and unstable invariant manifolds.

Approximations of stable (or unstable) manifolds are numerically computed to capture flow that asymptotically approaches an unstable periodic orbit in forward (or backward) time. An unstable periodic orbit is first discretized into $N_{m}$ states that are equally distributed along its arclength. Then, the *i*th state along the periodic orbit, $\boldsymbol{x}_{PO,i}$, is perturbed by a small magnitude *d* along each of the eigenvectors $\boldsymbol{v}_{S,i}$ and $\boldsymbol{v}_{U,i}$ of the monodromy matrix associated with the stable and unstable modes, respectively [6]. These states are propagated backward and forward in time, respectively [6]. The magnitude of the propagation time $t_{prop}$ is equal to a scalar multiple *c* of the time constant associated with the unstable eigenvalue $\lambda _u$, i.e., $t_{prop}=c T / \ln (|\lambda _{u}|)$ [38]. In this paper, $c=50$ for manifolds of $L_1$ Lyapunov, $L_2$ Lyapunov, and distant prograde orbits, whereas $c=30$ for the 4:5 resonant orbit used in Sect. 4.1 to ensure that paths leave the vicinity of the reference orbit and produce sufficiently diverse arcs, where possible. However, propagation is terminated early if the trajectory 1) impacts Neptune or Triton, approximated as spheres with radii of 24,764 km and 1352.6 km, respectively [30]; or 2) exceeds a distance of 10 nondimensional units from Neptune.

#### Curvature

Concepts from differential geometry are used to analyze and sample nonlinear trajectories. At any state along a trajectory, its position, velocity, and acceleration vectors are generally expressed in the rotating frame as $\boldsymbol{r}=[x,y,z]^T$, $\dot{\boldsymbol{r}}=[\dot{x},\dot{y},\dot{z}]^T$, and $\ddot{\boldsymbol{r}}=[\ddot{x},\ddot{y},\ddot{z}]^T$, respectively. When generated for a time interval $t\in [t_0, t_f]$, the trajectory traverses a distance in the rotating frame equal to the arclength [39]

3 $$\begin{aligned} s = \int _{t_0}^{t_f} ||\dot{\boldsymbol{r}}|| dt \end{aligned}$$

The instantaneous curvature $\kappa$ of the nonlinear trajectory is calculated as [39]

4 $$\begin{aligned} \kappa (t) = \frac{||\dot{\boldsymbol{r}}\times \ddot{\boldsymbol{r}}|| }{||\dot{\boldsymbol{r}}||^{3}} \end{aligned}$$

and possesses a singularity when the speed is zero. Maxima in the curvature, satisfying $\dot{\kappa }(\boldsymbol{x}) = 0$ and $\ddot{\kappa }(\boldsymbol{x}) < 0$, indicate locations along a trajectory where the shape is changing most rapidly. In the CR3BP, these curvature maxima tend to be located near the apses measured relative to meaningful reference points such as primaries or equilibrium points [40]. However, because curvature maxima do not require specification of a reference point, they supply an analytical criterion for automatically identifying geometrically meaningful points along trajectories throughout the Neptunian system.

#### Overview of Relevant Density-Based Clustering Algorithms

Clustering algorithms group similar members of a dataset while separating dissimilar members [41]. Similarity is typically assessed using a finite-dimensional description of each member of the dataset that is encoded into a feature vector. Although a wide variety of clustering algorithms exist, this paper leverages the following density-based clustering algorithms due to their focus on identifying a previously unknown number of clusters as members that form a sufficiently dense grouping in a feature vector space: 1) Density-Based Spatial Clustering of Applications with Noise (DBSCAN) [42] and 2) Hierarchical Density-Based Spatial Clustering of Applications with Noise (HDBSCAN) [43]. These algorithms have both been used to cluster spacecraft trajectories by their geometry in multi-body gravitational systems [27, 44, 45].

DBSCAN was developed by Ester et al. to identify groups of members that exist within neighborhoods that exceed a minimum density [42]. First, members of a dataset are categorized into one of the following three classes:

1. Core points possess at least $n_{pts}$ neighbors within a distance of $\epsilon$ in the feature vector space, labeled its $\epsilon$-neighborhood.
2. Border points exist in the $\epsilon$-neighborhood of a core point but do not possess at least $n_{pts}$ neighbors in their own $\epsilon$-neighborhood.
3. Noise points do not exist in the $\epsilon$-neighborhood of any core points.

Two core points that are labeled as density-connected can be reached by traversing a sequence of core points that lie within the $\epsilon$-neighborhoods of other core points. A density-connected set of core points forms a single cluster, along with their associated border points. Governed by the values of $n_{pts}$ and $\epsilon$, DBSCAN labels each member of the dataset as either belonging to a cluster or as noise.

HDBSCAN was developed by Campello, Moulavi, and Sander as an extension of DBSCAN [43]. First, the core distance of the *i*th member of a dataset, $d_{core}(\boldsymbol{f}_i)$, where $\boldsymbol{f}_i$ is a feature vector describing a dataset member, is defined as the distance to its $n_{core}$th nearest neighbor. Then, the mutual reachability distance between the *i*th and *j*th members of a dataset is calculated as

5 $$\begin{aligned} d_{reach} = \max (d_{core}(\boldsymbol{f}_i),d_{core}(\boldsymbol{f}_j),d(\boldsymbol{f}_i,\boldsymbol{f}_j)) \end{aligned}$$

This mutual reachability distance captures both the distance between the pair of members, $d(\boldsymbol{f}_i,\boldsymbol{f}_j)$, as well as their densities [43]. Then, a graph is formed to represent each member of the dataset as a node with weighted edges capturing their mutual reachability distance. A minimum spanning tree representation of this graph is then used to construct a hierarchical representation of all possible groupings of connected nodes as a function of the mutual reachability distance [43]. Finally, clusters are identified as groups of at least $n_{minsize}$ members that maximize stability across the hierarchy. Any members not assigned to a group are labeled as noise [43]. Based on a modification presented by Malzer and Baum [46], a quantity $\epsilon _{merge}$ specifies a minimum threshold on when members are split into multiple clusters. Through this process, HDBSCAN is governed by three parameters: $n_{minsize}$, $n_{core}$, and $\epsilon _{merge}$. Using these values, HDBSCAN labels each member of the dataset as either belonging to a cluster or as noise, but does not typically incorporate the concept of border points from DBSCAN.

#### Motion Primitives of Arcs Along Stable or Unstable Manifolds

Each of the $N_{m}$ trajectories that is generated to lie within a stable or unstable manifold of a periodic orbit is discretized into smaller arcs to support the extraction of a set of building blocks of motion. This approach follows the procedure presented by Gillespie, Miceli, and Bosanac [27]. Along each trajectory, the maxima in the curvature in the rotating frame are identified during numerical integration. These maxima in the curvature, as well as the initial condition when propagating forward in time, form the initial states for all arcs sampled from the trajectory. Then, the arcs are defined to span an additional $n_{\kappa ,max}=4$ maxima in the curvature and termination conditions, where applicable. Accordingly, two subsequent arcs that are sampled from the same trajectory shareup to four overlapping maxima in the curvature. For some arcs, this curvature-based definition corresponds to the completion of up to two revolutions around a moving center of curvature in the rotating frame. Compared with the proof of concept by Smith and Bosanac [26] that used apses to extract arcs along stable and unstable manifolds, the use of curvature maxima supplies a generalizable arc definition as a trajectory visits multiple regions of a system. In other cases, two maxima in the curvature along a trajectory can be located closer to each other as its shape transitions from convex to concave. Accordingly, these arcs can possess various arclengths and geometries but still be computed in a consistent manner.

Each continuous arc is sampled at a discrete set of states to support the construction of a finite-dimensional description. Specifically, each arc is sampled at $n_L = 13$ states: five states are located at each maximum in curvatureor initial and final states, and then two states are equally spaced in arclength between pairs of subsequent samples. Through this approach, the 13 states are sampled to capture the geometry of an arc with limited bias from variations in the speed or traversed distance across a diverse array of arcs. Furthermore, compared with the proof of concept by Smith and Bosanac [26] that sampled each arc only at its apses relative to a specified primary body, this sampling approach supplies a higher-fidelity approximation of each continuous arc and accommodates arcs revolving about various regions of a system.

The sampled states along a trajectory are used to define two feature vectors that support clustering by capturing 1) the shape of the trajectory and 2) the path traversed within the configuration space [45]. The shape-based feature vector $\boldsymbol{f}_v$ is defined as

6 $$\begin{aligned} \boldsymbol{f}_v = [\hat{\dot{x}}_1, \hat{\dot{y}}_1, \hat{\dot{z}}_1, ..., \hat{\dot{x}}_{n_L}, \hat{\dot{y}}_{n_L}, \hat{\dot{z}}_{n_L}]^T \end{aligned}$$

for a sequence of $n_L$ states, where $[\hat{\dot{x}}_i, \hat{\dot{y}}_i, \hat{\dot{z}}_i]$ is the velocity unit vector at the *i*th sample in the rotating frame. In addition, the position-based feature vector $\boldsymbol{f}_p$ is defined as

7 $$\begin{aligned} \boldsymbol{f}_p = [x_1, y_1, z_1, ..., x_{n_L}, y_{n_L}, z_{n_L}]^T \end{aligned}$$

where $[x_i, y_i, z_i]$ is the position vector at the *i*th sample in the rotating frame. Each feature vector produces a $3n_L$-dimensional description. Compared with the proof of concept by Smith and Bosanac [26], the use of two separate feature vectors as opposed to a single feature vector mitigates the challenges of scaling and combining distinct types of variables.

The feature vectors describing the arcs that are sampled from a stable or unstable manifold are clustered to identify groups of geometrically similar trajectories. To support accurately grouping a diverse set of trajectories by their geometry, clustering is performed in two steps: 1) HDBSCAN is used to generate a coarse grouping of trajectories by their shape, and then 2) each coarse group is refined using DBSCAN to produce groups of trajectories with a consistently similar shape and path. This clustering procedure was originally developed by Bosanac in Reference [45] and later adapted for motion primitives in Reference [27]. Compared with the proof of concept by Smith and Bosanac [26], this new clustering procedure 1) ensures that members of a cluster are geometrically similar, even when passing through various regions of a system with distinct sensitivities; 2) relies on a single, prespecified set of governing parameters; and 3) supports the recovery of clusters of various shapes and densities.

The arcs sampled from a stable or unstable manifold are coarsely grouped by their shape. First, the feature vector $\boldsymbol{f}_v$ is calculated using all $n_L=13$ states along the *i*th trajectory in the set. Repeating this process for all $N_m$ trajectories produces an $(N_{m}\times 39)$-dimensional matrix that is input to HDBSCAN to generate $N_{C}$ clusters and noise; if an arc is designated as noise, it is discarded. HDSBCAN is used in this step because it is useful when the number of clusters is not known a priori and the density and shape of those clusters might vary across the dataset. In this paper, the *hdbscan* Python library is employed during this clustering step [47].

During the initial coarse clustering, HDBSCAN is governed by three hyperparameters: $n_{minsize}$, $n_{core}$, and $\epsilon _{merge}$. The minimum cluster size is selected as $n_{minsize} = 5$, whereas $n_{core} = 4$ sets the neighborhood of each member to span the minimum cluster size, when added to the point itself [45]. These values are empirically selected to prioritize capturing localized variations across the dataset. The minimum threshold on the mutual reachability distance of members in distinct clusters is also selected using the heuristic $\epsilon _{merge} = 2\sqrt{13}\ \sin \left( 5^{\circ }\right)$ [48], i.e., the Euclidean distance between a sequence of 13 unit vectors that are separated by an angle of $10^{\circ }$. Finally, the Euclidean distance between two feature vectors is used to assess similarity.

Each coarse grouping of trajectories is refined to ensure that each cluster contains trajectories with a similar shape and path for their entire duration. This refinement procedure, originally developed by Bosanac [45], is modeled after convoy detection schemes in trajectory clustering [49]. To describe this process, consider one coarsely clustered group of $N_{cg}$ trajectories. The shape-based and position-based feature vectors $\boldsymbol{f}_v$ and $\boldsymbol{f}_p$ are evaluated at the *i*th state along each trajectory. The $(N_{cg}\times 3)$-dimensional dataset capturing the position-based feature vectors of the *i*th state along all the trajectories in the coarse group is input to DBSCAN. The output is clusters of trajectories where the *i*th state is density-connected to at least $n_{pts}$ other trajectories in the position-based feature vector space. Repeating this clustering step in each of the position and shape-based feature vector spaces at all 13 samples produces 26 independent clustering results. If at least $n_{minsize}$ trajectories are consistently grouped together in all 26 clustering results, they form a refined cluster. At this stage, cluster refinement can also split coarse groups into multiple clusters or remove outliers. Repeating this process for all $N_{C}$ coarse groups produces a new set of $N_{R}$ refined clusters and noise; any trajectories designated as noise at this stage are also discarded. In this paper, the MATLAB implementation of DBSCAN is used for clustering [50].

During cluster refinement, DBSCAN is governed by two hyperparameters: $n_{pts}$ and $\epsilon$. In this paper, $n_{pts}=n_{core}=4$ is selected to define the span of each neighborhood to be consistent with the coarse grouping step. In addition, the neighborhood radius is set equal to the following heuristic: $\epsilon =(n_{pts}+1)\max (e,\epsilon _{threshold})$ where $\epsilon _{threshold}$ is $2\sin \left( 2.5^{\circ }\right)$ and $10^{-3}$ in the shape-based and position-based feature vector spaces, respectively, and *e* is the $n_{minsize}$-largest distance between each member and its nearest neighbor. Although slightly modified from Reference [45], this heuristic is observed to limit bias from any outliers in the coarse group while assigning sufficiently similar trajectories to each refined cluster. However, further improving this heuristic is an area of ongoing work.

As presented by Smith and Bosanac, motion primitives are extracted as the medoid of each cluster [25]. The medoid of a cluster possesses the minimum cumulative distance from all other members [41]. In this paper, the medoid is calculated using the 39-dimensional position-based feature vectors describing the trajectories in a cluster. Then, up to 50 additional trajectories in the cluster are extracted to coarsely represent the region of existence for each primitive and be approximately equally distributed in the position-based feature vector space. Each primitive *P* and its coarsely approximated region of existence $\mathcal {R}(P)$ are stored in the motion primitive library.

**Fig. 1:**
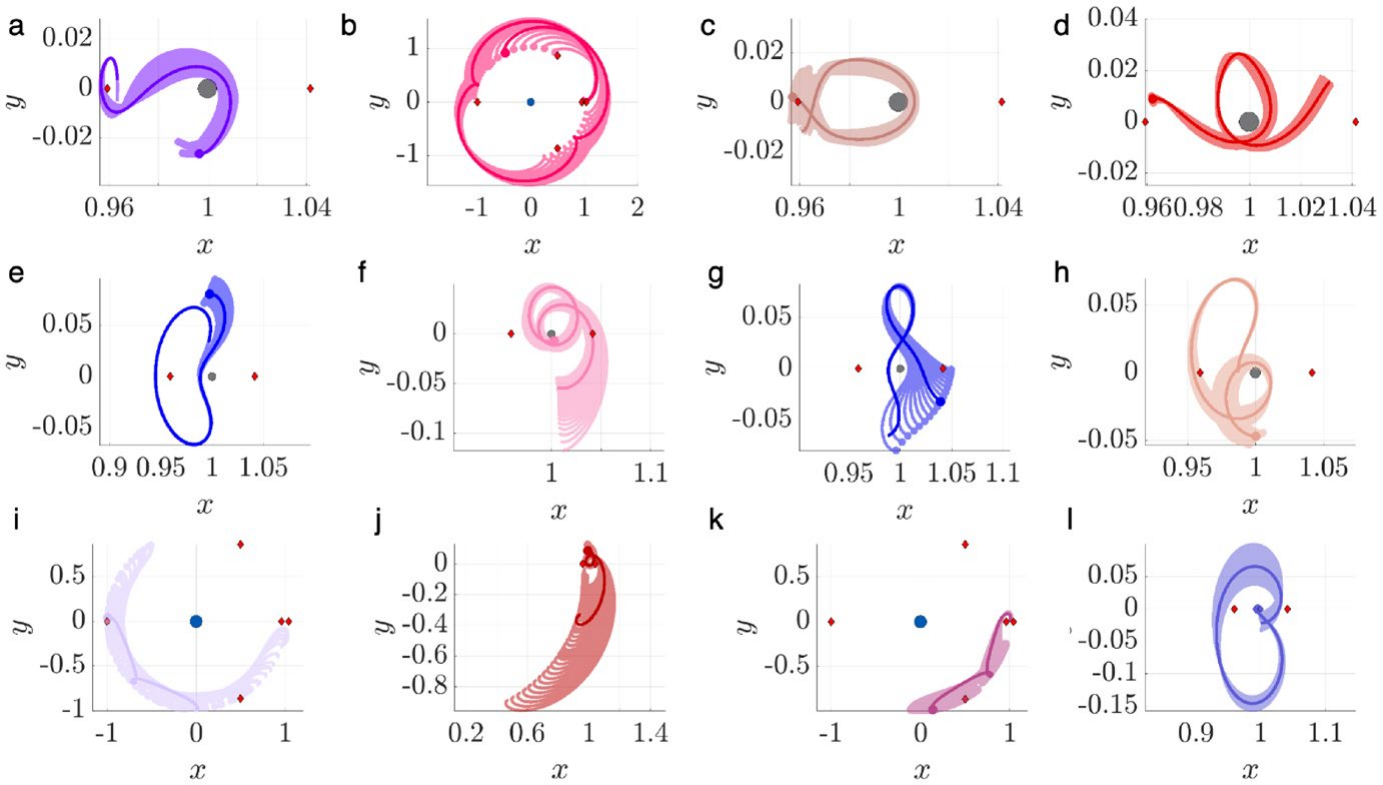
Selected motion primitives (thick curves) and their regions of existence (translucent) for stable and unstable manifolds associated with $L_1$ Lyapunov orbits at $C_J =[2.9981, 3.0037, 3.01400]$

To demonstrate this procedure, consider motion primitives extracted from arcs along the stable and unstable manifolds of three $L_1$ Lyapunov orbits at $C_J=[2.9981, 3.0037, 3.01400]$. Figure 1 displays 12 of the 518 generated primitives, plotted in uniquely colored thick curves in the rotating frame, along with their associated regions of existence, indicated by additional trajectories that are plotted as translucent. The circle marker along each motion primitive indicates its initial condition, showing the direction of motion. In addition, the blue and gray circles indicate Neptune and Triton, scaled by their equatorial radii of 24,764 km and 1,352.6 km [30], respectively, whereas the red diamonds locate selected equilibrium points. This same figure configuration is used throughout this paper when visualizing selected motion primitives or trajectories. These examples demonstrate that each region of existence is spanned by geometrically similar trajectories, and the motion primitive summarizes their common geometry. Furthermore, distinct motion primitives summarize groups of arcs with sufficiently different geometries or paths.

#### Motion Primitives of Periodic Orbit Families

A group of geometrically similar periodic orbits is defined as a continuous segment of a family with a similar evolution of the curvature along each orbit. This approach, previously presented by Gillespie, Miceli, and Bosanac [27], requires that two geometrically similar trajectories possess similar time histories of curvature with the same number of stationary points. Through this approach, a straightforward analytical criterion is used to separate geometrically distinct periodic orbits along a family. Accordingly, this approach improves upon Smith and Bosanac’s prior proof of concept [26] by assigning every sampled periodic orbit to a group, eliminating the reliance on governing parameters, eliminating the challenges of discovering dense or separated groupings of members of a continuous family, and reducing the required computational effort.

Groups of geometrically similar periodic orbits are extracted by detecting changes in the number of maxima in the curvature along neighboring, discretely sampled members along the family. Along each periodic orbit, the curvature is calculated using Eq. (4) at each state that is output from the numerical integration scheme. Then, the number of local maxima in the curvature along the trajectory is calculated as the number of peaks in $\kappa (t)$. Stepping from the first member along the family, new groups of geometrically similar periodic orbits are formed whenever the number of maxima in the curvature changes between neighboring members.

A motion primitive and its region of existence are computed to summarize each group of geometrically similar periodic orbits along a family. First, the motion primitive *P* is extracted as the periodic orbit with an arclength closest to the mean value across the group. Then, up to 20 additional orbits that are approximately evenly distributed in the position-based feature vector space are extracted to coarsely represent the associated region of existence $\mathcal {R}(P)$.

**Fig. 2:**
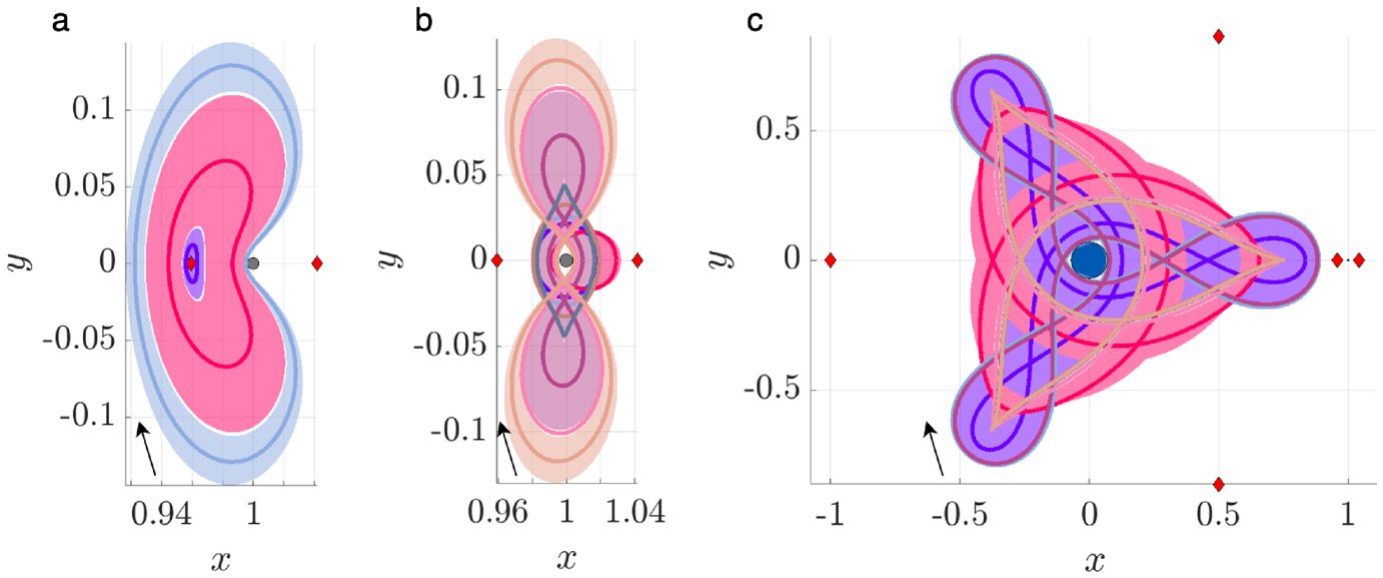
Selected motion primitives (thick curves) and their regions of existence (translucent shading) for the **a** $L_1$ Lyapunov orbit family, **b** distant prograde orbits, and **c** a 3:1 resonant orbit family

To demonstrate this process, consider motion primitives extracted to summarize selected planar periodic orbit families. Each subfigure of Fig. 2 displays motion primitives and the associated regions of existence for the following families: a) $L_1$ Lyapunov orbits, b) DPOs, and c) 3:1 resonant orbits. These subfigures are plotted with a similar configuration to Fig. 1. However, each subfigure displays all the primitives and their regions of existence along each family of periodic orbits. This figure demonstrates that each motion primitive summarizes the geometry of each segment along the selected families. Furthermore, distinct motion primitives capture both obvious and subtle geometric changes along each family of periodic orbits.

### Step 2: Motion Primitive-Informed Graph

A motion primitive-informed graph is constructed to discretely approximate a segment of the continuous solution space. This section presents a new definition of a motion primitive-informed graph, substantially building upon the original proof of concept by Smith and Bosanac [25]. The most significant improvements include: 1) updating the definition of sequential composability; 2) representing each motion primitive using multiple nodes to encode traversing segments of the associated arcs; 3) adding edges only between nodes representing segments of primitives that exist nearby in the configuration space; 4) updating the edge weight definition to reflect changes in the velocity direction; and 5) incorporating path constraints early in the trajectory design process. These improvements result in a better estimate of the sequential composability of two primitives; the capability to encode multiple intersections between two primitives at distinct locations; and a higher likelihood that a primitive sequence predicts the existence of a nearby continuous, constrained trajectory.

#### Selecting Motion Primitives for the Graph

To begin the graph construction process, candidate motion primitives are selected from the library computed in Step 1. In this paper, candidate motion primitives are selected in groups by specifying the fundamental solutions that they summarize. For instance, in the trajectory design example used in this section, the designer may include all motion primitives summarizing the $L_1$ and $L_2$ Lyapunov orbit families as well as arcs along their stable and unstable manifolds. During the selection process, the designer may incorporate any knowledge of a desired itinerary, bounds on the Jacobi constant, or geometric characteristics.

The selected motion primitives and their regions of existence are compared to all specified path constraints. These path constraints may include, for example, a minimum or maximum distance from a body, a cone angle constraint, or a keep-out zone. However, because the motion primitive graph is only a discrete approximation of the solution space, a small margin is incorporated into each constraint at this stage. For instance, if the minimum allowable altitude above Triton is equal to 500 km, a designer-specified margin of 200 km could be applied to modify this constraint to 300 km during the primitive selection process. Of course, during trajectory corrections and optimization at later stages of the design process, this margin is removed.

After comparing the motion primitives and their regions of existence to all path constraints, the primitive is either added to the graph, modified, or discarded. If a motion primitive and every arc spanning its region of existence satisfy every path constraint, it is used in the graph construction process. Alternatively, if the motion primitive and all arcs spanning its region of existence violate the constraint, the motion primitive is not added to the graph. If at least one but not all arcs violate the constraint, the region of existence is resized to contain only members that satisfy all the path constraints. In the case that the motion primitive violates at least one constraint, the motion primitive is recalculated using all other arcs within the region of existence that satisfy every path constraint. Any primitives that satisfy all constraints are labeled as feasible motion primitives.

#### Identifying Sequentially Composable Motion Primitives

**Fig. 3:**
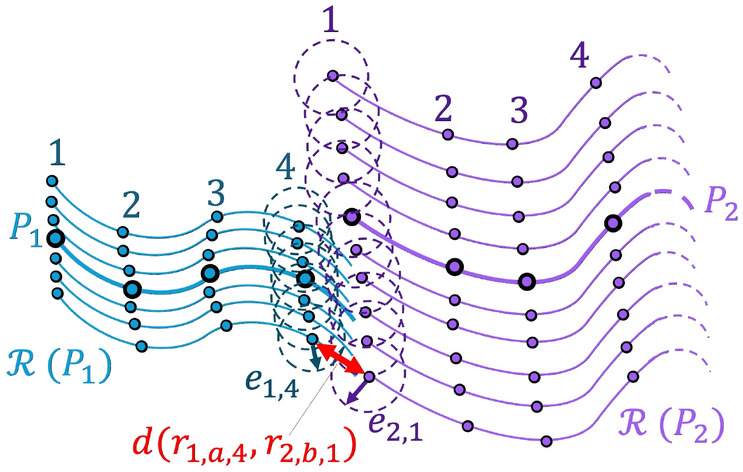
Conceptual depiction of sequential composability assessment between two primitives and trajectories spanning their regions of existence

Two motion primitives are designated as sequentially composable if the sampled states along trajectories within their regions of existence are closely located in the configuration space with constrained differences in the velocity. If they are sequentially composable, the pair of primitives is expected to predict the existence of a nearby continuous trajectory with a similar geometry, potentially with a single change in velocity implemented via an impulsive maneuver. To describe the assessment of sequential composability in the following paragraphs, Fig. 3 conceptually depicts two motion primitives and a subset of trajectories that span their regions of existence: trajectories in $\mathcal {R}(P_1)$ are displayed in blue whereas trajectories in $\mathcal {R}(P_2)$ are colored purple. In each case, the motion primitive is highlighted by a thick curve, and the samples along the primitives are represented as black circles.

The region of the configuration space spanned by the *i*th samples along all trajectories in the region of existence of a primitive is coarsely approximated. Consider the region of existence associated with the blue primitive, i.e., $\mathcal {R}(P_1)$ in Fig. 3. The fourth sample along each of the seven blue trajectories is isolated, and a circle is centered around each of the seven samples in the configuration space. The radius of every circle is calculated as the average distance between each of these seven samples and their nearest neighbor in the configuration space, multiplied by a user-defined constant $c_c=1.5$ to add margin. For this example, this radius is labeled ${e}_{1,4}$, where the first subscript identifies primitive $P_1$ and the second subscript identifies the fourth sampled state. The collection of these circles approximates the region of the configuration space spanned by the fourth sampled state along the purple trajectories in $\mathcal {R}(P_1)$.

To determine whether two primitives are sequentially composable, the regions of the configuration space spanned by their sampled states are compared. As depicted in Fig. 3, the distance between the fourth sampled state along the *a*th trajectory within $\mathcal {R}(P_1)$ (blue) and the first sampled state along the *b*th trajectory within $\mathcal {R}(P_2)$ (purple) is calculated in the configuration space and denoted as $d(\boldsymbol{r}_{1,a,4},\boldsymbol{r}_{2,b,1})$; in each position vector, the subscripts indicate the primitive, trajectory, and sampled state, respectively. Using the triangle inequality, if these distances calculated for any combination of *a* and *b* satisfy the condition $d(\boldsymbol{r}_{1,a,4},\boldsymbol{r}_{2,b,1}) \le (e_{1,4} + e_{2,1})$, the circles centered on the associated states overlap. Accordingly, the fourth sampled state along the *a*th blue trajectory is considered to be sufficiently close to the first sampled state along the *b*th blue trajectory in the configuration space. Thus, primitives $P_1$ and $P_2$ are considered sequentially composable at those sampled states, with an impulsive maneuver changing the velocity vector as needed. This approach is useful for determining the composability of states located in the interior of the region of existence. However, depending on the average distance between the members of the primitive’s region of existence, the radius *e* around the boundary nodes could overestimate the composability. Addressing this limitation is a subject of ongoing work.

To support the trajectory design process, a velocity-based constraint is also used in assessing whether two primitives are sequentially composable. In this paper, the angle between the velocity vectors must be below a specified threshold $\theta _{max}$, consistent with the states possessing a sufficiently similar direction of motion. Using the sampled states in the previous paragraph as an example, this constraint is equal to:

8 $$\begin{aligned} \angle (\hat{\boldsymbol{v}}_{1,a,4},\hat{\boldsymbol{v}}_{2,b,1}) \le \theta _{max} \end{aligned}$$

In this paper, $\theta _{max}=30^{\circ }$. An additional constraint on the magnitude of the velocity difference based on maneuver magnitude limits is not incorporated into the general sequential composability condition in this paper because 1) an upper bound may vary as spacecraft parameters or mission requirements in a specific trajectory design scenario evolve, and 2) coarse approximations of velocity differences between primitives may not reflect actual maneuver requirements after corrections and optimization. However, such a constraint could also be included in the sequential composability definition in applications of this framework, if desired.

#### Defining the High-Level Graph Structure

The high-level structure of the motion primitive-informed graph is defined by coarsely specifying the order and potential connectivity of the motion primitives. In this paper, the feasible motion primitives are organized into three or more sets that correspond to the order in which they can be connected within the graph and, therefore, composed to eventually produce initial guesses. These sets are defined as follows:

1. Initial set: motion primitives or arcs defining the possible initial conditions.
2. Transfer set/s: all motion primitives that could be used to connect the boundary conditions. These motion primitives could be organized into one or more sets with intermediate waypoints to constrain the itinerary, if desired.
3. Target set: motion primitives or arcs defining the possible target conditions.

The potential connectivity between these sets must be specified. In this paper, a directed connection is allowed 1) from any primitives in the initial set to the transfer set and 2) from any primitives in the transfer set to the target set. Next, the potential connectivity of primitives within each set is also specified. For instance, motion primitives within the transfer set may be connected to any other primitives within the same set. However, the designer may choose to prohibit any motion primitives in the initial or target set from being connected to other primitives in the set, consistent with their use as boundary conditions. Of course, the designer could select a different high-level graph structure based on their specific trajectory design problem.

**Fig. 4:**
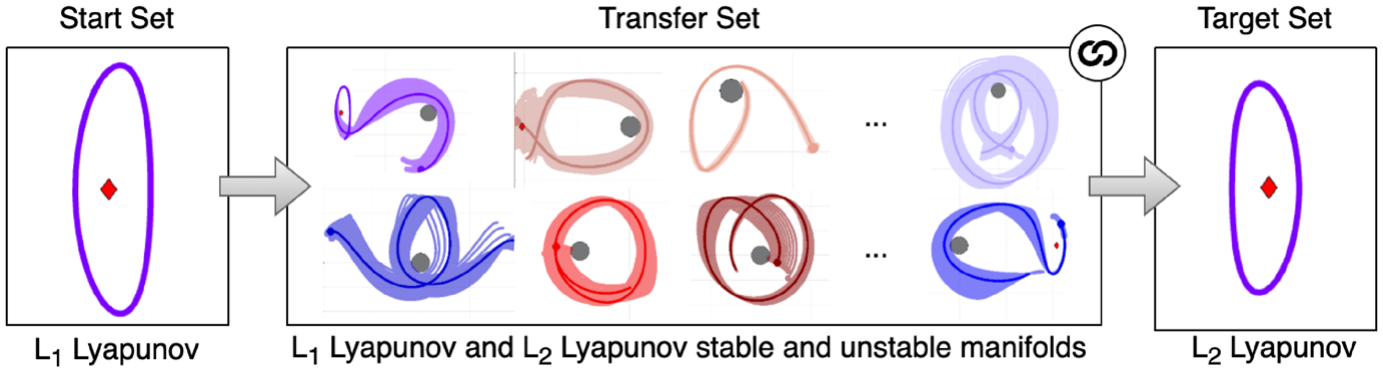
Example of high-level graph structure for designing a transfer between an $L_1$ Lyapunov orbit at $C_J = 3.01400$ and $L_2$ Lyapunov orbit at $C_J = 3.01377$ in the CR3BP

An example of a high-level graph structure is depicted in Fig. 4 for the example transfer design scenario used in this section. The initial set contains an $L_1$ Lyapunov orbit at $C_J = 3.01400$, whereas the target set contains an $L_2$ Lyapunov orbit at $C_J = 3.01377$. The transfer set contains 75 motion primitives from the unstable and stable manifolds of the Lyapunov orbits used to define the boundary conditionsthat remain near Triton. In this example, the primitives in the left or middle sets can be connected with the primitives in the set immediately to its right, if the pair of primitives is sequentially composable. The symbol at the top right of the transfer set block indicates that the primitives within the set can be connected to any other primitive in the set. The initial state, integration time, and Jacobi constant of all primitives included in this set are provided as supplementary data in the form of a text file (Online Resource 2).

#### Constructing the Nodes of the Graph

Each motion primitive that is summarized by $N_s$ discretely sampled states contributes $N_s$ nodes to the graph. For motion primitives that are generated to summarize arcs along stable or unstable manifolds, the geometry-based sampling strategy from Step 1 is used to generate $N_{s,m}=13$ sampled states. However, a motion primitive that summarizes a segment of a periodic orbit family is sampled at maxima in curvature and at five points equally distributed along the arclength between each subsequent pair of maxima; five intermediate samples are used to better represent resonant orbits that feature long arcs between subsequent maxima in the curvature. The number of sampled states $N_{s,p,i}$ of the *i*th periodic orbit primitive depends on the associated number of curvature maxima. Accordingly, a motion primitive-informed graph that includes $M_m$ motion primitives that are generated from arcs along stable or unstable manifolds and $M_p$ primitives that summarize periodic orbits is composed of $M_mN_{s,m} + \sum _{i=1}^{M_p} N_{s,p,i}$ nodes. Each of these nodes is associated with a specific set of sampled states along the region of existence associated with one motion primitive, e.g., one node represents the fourth sampled states along trajectories in $\mathcal {R}(P_1)$.

#### Constructing the Edges of the Graph

Directed, weighted edges are added between pairs of nodes to encode allowable transitions between their associated states via natural motion or impulsive maneuvers. These edges connect nodes along the same primitive as well as nodes along distinct, sequentially composable primitives. Each edge is weighted by positive scalar values that reflect the change in the velocity direction between the two nodes.

Directed, weighted edges are added between nodes from the same primitive. A directed edge is added from the node associated with the *i*th sampled state to the node associated with the $(i+1)$th state along the same motion primitive for integers $i\in [1,N_{s})$. These edges are assigned a weight of $q_{i,i+1} = 10^{-15}$ to reflect that the nodes are connected via natural motion. To support encoding the edges and their weights into an adjacency matrix, these edge weights are assigned a small, positive value to avoid being mistaken for a zero, which indicates that two nodes are not connected.

Directed edges are also added between nodes along distinct, sequentially composable primitives that satisfy the composability conditions, follow the high-level graph structure, and satisfy all constraints. Consider two nodes, *i* and *j*, that correspond to sequentially composable states along distinct primitives. The positive weight $q_{i,j}$ of an edge connecting these nodes reflects the minimum change in velocity direction between all sequentially composable combinations of the *i*th and *j*th sampled states along trajectories within the region of existence of each primitive.

This edge weight is calculated as

9 $$\begin{aligned} q_{i,j} = \max \left[ \min _{a,b} \left( 1 - \hat{\boldsymbol{v}}_{i,a}\cdot \hat{\boldsymbol{v}}_{j,b}\right) ,10^{-15}\right] \end{aligned}$$

where the first subscript of the velocity unit vector identifies the node, whereas the second subscript indicates the trajectory within the associated region of existence. By trigonometry, the magnitude of the difference between two velocity vectors with a fixed angular separation increases as their speeds increase. Using the change in velocity direction in Eq. (9) mitigates this influence of the speed on the edge weight as the primitives pass through distinct regions of the system. Accordingly, this edge weight supports 1) recovering primitive sequences that may potentially pass close to either primary but still possess relatively smooth changes in the velocity direction, and 2) recognizing that maneuvers may be necessary to change the speed but are overestimated prior to corrections and optimization.

Each edge is also accompanied by a list of the representative trajectories in the regions of existence that satisfy the sequential composability conditions at nodes *i* and *j*. For the example in Sect. 3.2.2, the edge connecting node four along the blue primitive to node one along the purple primitive would be accompanied by the list [*a*, *b*]. This information is used during the graph search process, as described in the next section.

### Step 3: Generate Sequences of Motion Primitives

Consistent with path planning literature, a motion primitive graph is typically searched to produce a sequence of composable motion primitives that form complex paths [19]. In their proof of concept, Smith and Bosanac used a depth-first searchalgorithm to generate primitive sequences from a motion primitive graph [25]. However, this paper presents a modified *k*-best paths search algorithm that leverages A* [51] and Yen’s algorithm [52] to generate multiple sequences of motion primitives that possess the lowest cumulative changes in the velocity direction. Through this approach, longer and more diverse motion primitive sequences are generated more efficiently.

#### Overview of Relevant Graph Search Algorithms

Consider a graph composed of a set of nodes and directed, weighted edges. The shortest-path problem involves generating an optimal path through the graph, i.e., a sequence of connected nodes, from an initial node to a target node that minimizes the cumulative edge weights. An additional $k-1$ suboptimal paths may also be of interest to supply alternative options. Although a variety of graph search algorithms exist to solve these problems, this paper uses A* [51] and Yen’s algorithm [52].

The A* graph search algorithm, first presented by Hart, Nilsson, and Raphael in 1968, generates the optimal path between a single initial node and a single goal node [51]. At the *i*th node, A* explores the neighboring nodes by computing the cost $f_{i,j} = g_{i,j}+ h_{j}$, where $g_{i,j}$ is the cost from the current node *i* to the next node *j*, and $h_{j}$ is the expected cost from the next node *j* to the target node [51]. The incomplete paths, the cost along the incomplete paths labeled $f_t$, and their cumulative edge weights, labeled as $g_t$, are stored in a queue. After the closest neighbors of the current node have been explored, the incomplete path that minimizes the total path cost is selected for further exploration. This process repeats until the target node has been reached. If the selected heuristic satisfies the condition $h_j \le h^*_j$, where $h^*$ is the true cost to traverse the graph from the selected node to the target node, A* is guaranteed to return the optimal path. Furthermore, by including this heuristic, A* produces the optimal path with lower computational complexity and in less time than the well-known Dijkstra’s algorithm [53].

Yen’s algorithm identifies *k* paths without any cycles through a directed, weighted graph [52]. Once the optimal solution within a weighted graph has been identified with a selected search algorithm, Yen’s algorithm identifies the next $k-1$ best paths through a set of subgraphs. Each subgraph is a modification of the original graph: each of the $n_e$ edges between connected nodes of the most recently computed best path is individually removed from the original graph to produce $n_e$ subgraphs. Then, the selected search algorithm is used to generate the optimal path through each subgraph starting from the end of the root path, i.e., the sequence of nodes composing the best path up to the removed edge. From this set of new paths, the path with the lowest cumulative edge weight is saved as the latest best path. This process is repeated from the latest best path until the *k* best or all possible paths have been identified. As *k* increases or all possible paths have been calculated, these *k* best paths approach the globally best solutions. In astrodynamics, Yen’s algorithm was previously used by Bruchko and Bosanac to search a graph representation of a roadmap of randomly-sampled states connected by natural and impulsive maneuver-enabled arcs to generate initial guesses for geometrically distinct transfers in the Earth-Moon CR3BP [18].

#### Searching the Motion Primitive-Informed Graph

In this paper, the cost function $f_{i,j}$ is calculated using the edge weight $q_{i,j}$ defined in Step 3. In addition, the heuristic $h_{j}$ is defined as the actual cost required to reach the target node from node *j*, ensuring that the optimal path through the graph is identified. This value is calculated using the well-known Dijkstra’s algorithm, which is equivalent to A* with a heuristic of zero [54].

To generate a primitive sequence, the A* algorithm is slightly modified. The algorithm used in this paper is summarized in Algorithms 1 and 2. To encourage the generation of diverse paths in the search process while identifying node sequences with relatively low edge weights, this paper modifies the logic used by A* to select the neighboring nodes to explore in lines 5–10. The first modification in lines 5–6 of Algorithm 1 focuses on constraining the traversability of each primitive. Consider an incomplete path composed of four nodes through the graph: the first three nodes lie along $P_1$; the fourth node lies along $P_2$; and the third trajectory in $\mathcal {R}(P_1)$ is sequentially composable with both the fourth and fifth trajectories in $\mathcal {R}(P_2)$. The next, neighboring nodes to explore can either correspond to 1) subsequent states along $P_2$ or 2) lie along a different primitive that is sequentially composable with the fourth or fifth trajectories in $\mathcal {R}(P_2)$ at a subsequent sampled state. The second condition ensures that the entry point to the region of existence associated with a primitive is mapped to possible departure points in a manner that is consistent with the flow of trajectories with a specific geometry. From this subset of neighboring nodes, Algorithm 2 further constrains neighbor selection as follows:

- Line 3 ensures that more than one sequential node along a primitive is selected, producing an arc with a nonzero integration time.
- Line 5 supports increasing the diversity of paths after the initial condition, although the associated node may be suboptimal with respect to $f_t$.
- Line 8 prioritizes the natural flow along a primitive while ensuring the least expensive path is explored.
- Line 10 ensures that an additional *r* incomplete paths are added to the queue for future consideration, improving exploration of the solution space.

This procedure identifies a sequence of connected nodes and edges that minimizes the cumulative edge weight. However, the limitation of this approach is that storing and using traversability information increases the computational effort required to search a graph.

**Algorithm 1:**
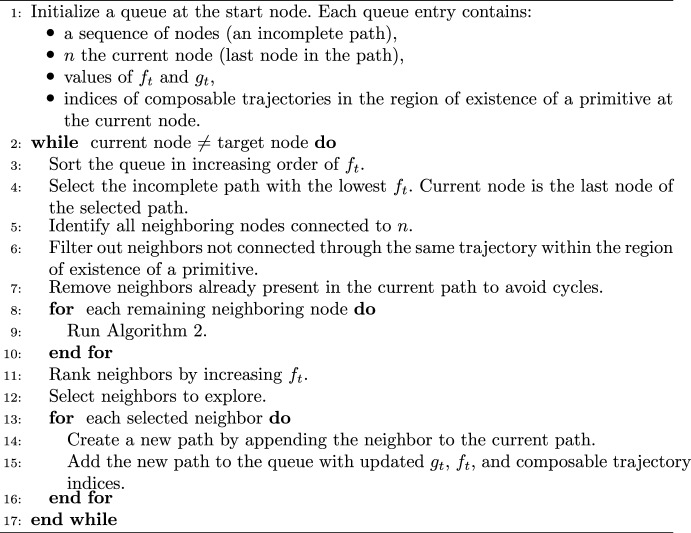
Modified A* for Searching a Motion Primitive-Informed Graph

**Algorithm 2:**
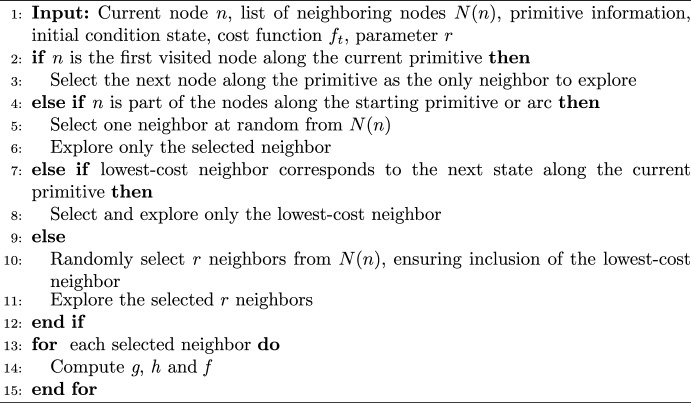
Neighbor Selection Strategy

Each path through the graph is translated into its associated primitive sequence. Consider a sequence of ten nodes: the first four nodes are associated with states along $P_1$, the next three nodes are associated with states along $P_2$, and the final three nodes are associated with states along $P_3$. This node sequence is translated into the primitive sequence $P_1-P_2-P_3$. Because distinct motion primitives that are derived from the same fundamental solution are generated to be geometrically distinct, unique primitive sequences can produce geometrically distinct initial guesses for trajectories.

To identify an additional $k-1$ diverse primitive sequences, Yen’s algorithm is slightly adapted to the characteristics of the motion primitive-informed graph. The pseudocode of the algorithm is presented in Algorithm 3. The modification of Yen’s algorithm in line 7 removes all edges between nodes along the same primitive, rather than a single edge between subsequent nodes, when constructing each subgraph. Accordingly, if the latest best path is composed of $N_n$ nodes that correspond to a sequence of $N_p$ motion primitives, $N_p - 2$ subgraphs would be created at each iteration of Yen’s algorithm since the first and last primitives are never removed. Moreover, paths searched on the subgraphs are not constrained to start from the root of the last best path, as in the original Yen’s algorithm [52]; this modification offers more flexibility in recovering an optimal path that may be geometrically different from the latest best path. Another difference from the traditional implementation of Yen’s algorithm is the emptying of the list containing the paths obtained from the subgraphs after each iteration. This choice promotes the selection of a more diverse array of paths at the expense of path optimality. This search algorithm produces *k* diverse, yet relatively low-cost paths through the graph that connect the initial and target nodes.

**Algorithm 3:**
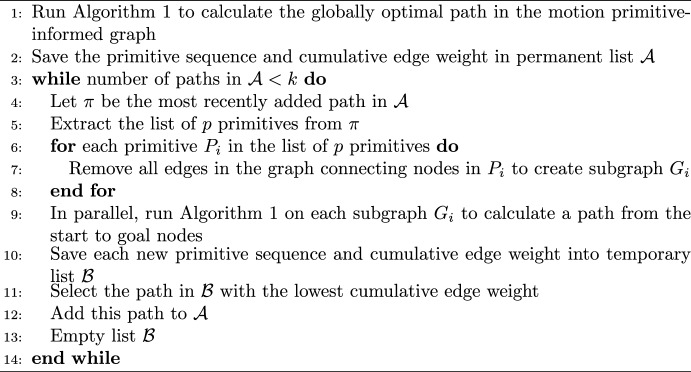
k-Best Paths Search Algorithm

### Step 4: Constructing an Initial Guess for a Trajectory

Each sequence of motion primitives is used to generate an initial guess for a trajectory. Similar to Step 2, this step is formulated using a refinement graph that captures the potential sequential composability of each primitive within the sequence, as well as the geometrically similar arcs that are sampled across their associated region of existence. This refinement graph is then searched to generate the sequence of arcs that minimize the position discontinuity and remove overlap between arcs along sequential primitives. In their early proof of concept, Smith and Bosanac used a sequential morphing and trimming approach to greedily select pairs of arcs from within the region of existence of neighboring primitives to minimize the full state discontinuity [25]. However, the new approach presented in this paper supplies a globally optimal, refined initial guess that resembles the original primitive sequence.

#### Defining the High-Level Graph Structure

**Fig. 5:**
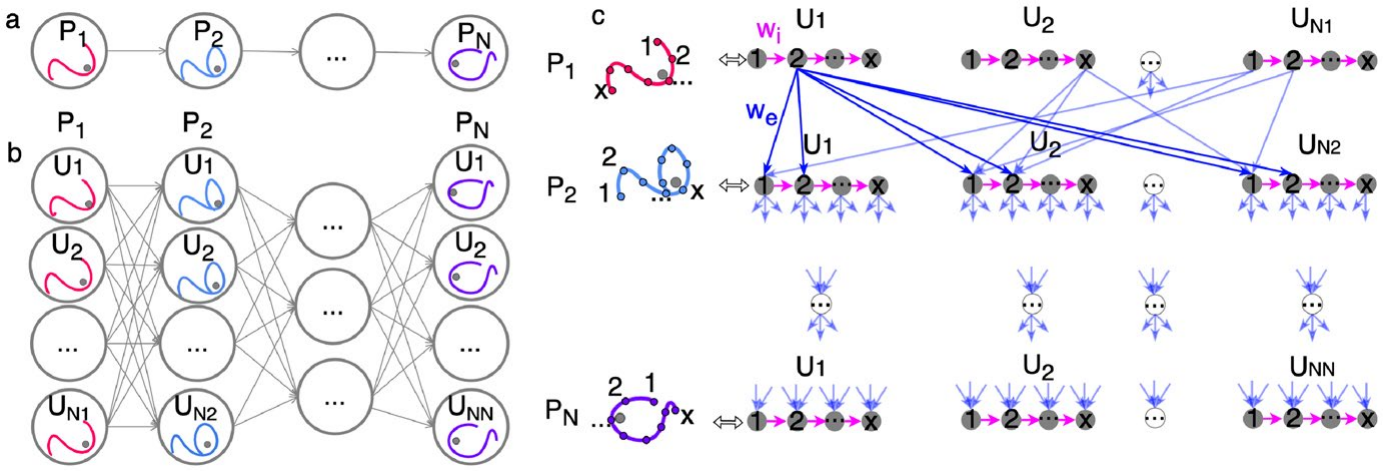
Constructing a refinement graph to support generating an initial guess from a motion primitive sequence

A high-level graph structure is first defined. Consider a sequence of $N_p$ motion primitives, $P_1-P_2-... -P_N$, as depicted in Fig. 5a. The *i*th motion primitive and the $U_i$ arcs that represent $\mathcal {R}(P_i)$ are represented using $U_i+1$ nodes that form the *i*th layer of the high-level graph structure, as depicted in Fig. 5b. This process is repeated for all $N_p$ motion primitives to produce $\sum _{i=1}^{N_p}(U_i+1)$ nodes that are arranged into $N_p$ layers. When $i<N_p$, each node in the *i*th layer of the high-level graph structure is connected to all nodes in the $(i+1)$th layer using unidirectional edges. However, nodes within the same layer are not connected. Through this formulation, the graph structure reflects all possible sequences of arcs that could be composed to geometrically resemble the motion primitive sequence generated in Step 3.

#### Constructing the Graph

The nodes of the refinement graph are defined as the states sampled along each arc captured in the high-level graph structure, consistent with the approach presented in Step 2. Consider the *j*th arc within the *i*th layer of the high-level graph structure. This arc is sampled using $N_{s,i,j}$ states, following the procedure outlined in Sect. 3.2.4. Each of these $N_{s,i,j}$ states contributes a single node to the refinement graph. Repeating this procedure across all arcs captured in the high-level graph structure results in the refinement graph consisting of $\sum _{i=1}^{N_p}(\sum _{j=1}^{U_i+1}N_{s,i,j})$ nodes.

Directed, weighted edges are added between nodes that are sampled from the same arc. Consider the $N_{s,i,j}$ nodes in the refinement graph that are generated by sampling the *j*th arc from the *i*th layer of the high-level graph structure. The *k*th node is connected to the $(k+1)$th node, for $k=[1,N_{s,i,j}-1]$ using a directed edge with a weight of $w_{k,k+1}=10^{-15}$ to reflect that these nodes are connected via natural motion. These edges are depicted with magenta arrows in Fig. 5c and labeled $w_i$.

Composable nodes from arcs within the region of existence of sequential primitives are connected via directed, weighted edges. Specifically, edges are only added between nodes in the *i*th and $(i+1)$th layer of the high-level graph structure if their associated states satisfy the sequential composability condition defined in Step 2. However, an edge that connects node *a* to node *b* in the refinement graph is weighted by the normalized discontinuity between their position vectors because the focus when constructing an initial guess is on reducing position discontinuities between arcs. This edge weight is calculated as

10 $$\begin{aligned} w_{a,b} = \frac{\Delta {r}_{a,b} - \Delta {r}_{min}}{\Delta {r}_{max} - \Delta {r}_{min}} \cdot [1 - 10^{-10}] + 10^{-10} \end{aligned}$$

where $\Delta {r}_{a,b}$ is the distance between nodes *a* and *b*, $\Delta {r}_{max}$ and $\Delta {r}_{min}$ are the maximum and minimum distances between all the composable nodes within $\mathcal {R}(P_{i})$ and $\mathcal {R}(P_{i+1})$. Normalizing the position difference between two nodes to within the range $w_{a,b} \in [10^{-10},1]$ mitigates biasing due to the size of the region of existence and the spacing between arcs sampled to span this region while ensuring that the edge weight remains positive. Figure 5c depicts these edges using blue arrows and labeled $w_e$.

#### Searching the Graph

The refinement graph is searched to generate a path that minimizes the cumulative edge weights. This path corresponds to a sequence of nodes along arcs that geometrically resemble a portion of each motion primitive in a sequence while minimizing position discontinuities between arcs. This search is performed using the A* algorithm with a minor modification to ensure that each path consists of at least two nodes from an arc within the region of existence associated with each motion primitive. Thus, each initial guess features a finite-length arc resembling each primitive in the sequence.

#### Examples of Initial Guesses

**Fig. 6:**
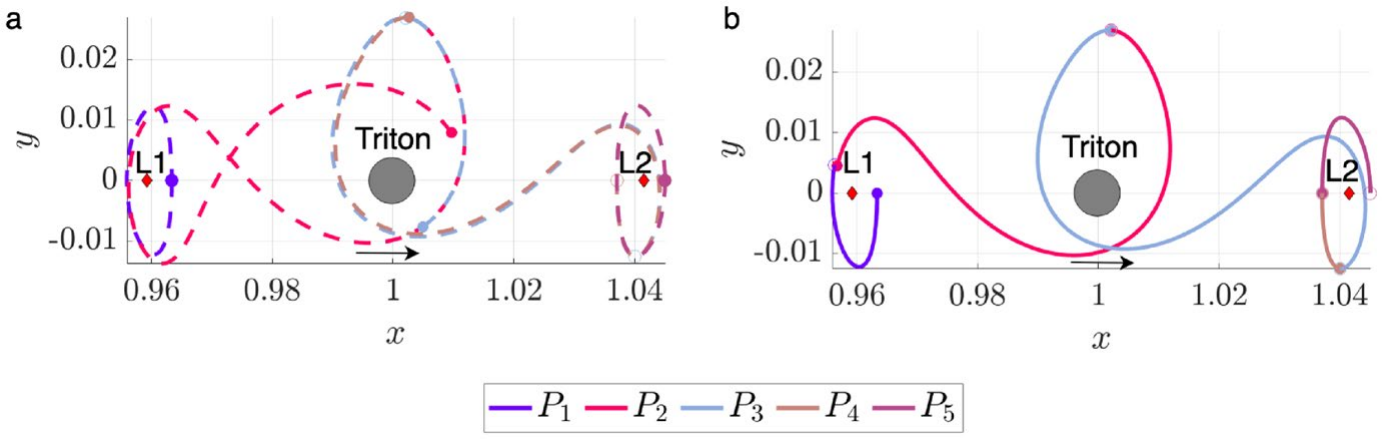
**a** Primitive sequence generated in Step 3 and **b** associated initial guess generated in Step 4

To demonstrate the procedure for generating an initial guess, consider the transfer example from an $L_1$ Lyapunov orbit at $C_J = 3.01400$ to an $L_2$ Lyapunov orbit at $C_J = 3.01377$. In this example, the final motion primitive graph obtained from the high-level itinerary in Fig. 4 is composed of 1003 nodes and 12,058 edges. Searching this graph for the optimal path produces a sequence of five primitives that are displayed in Fig. 6a using dashed lines, with each unique color corresponding to a different primitive. The refinement process described in this section produces the initial guess in Fig. 6b using solid curves. The start and end states along each arc sampled from the region of existence of a motion primitive are indicated by filled and open circle markers, respectively. This initial guess minimizes position discontinuities and overlap between subsequent arcs, while still retaining the geometry of the connected subsets of each motion primitive.

**Fig. 7:**
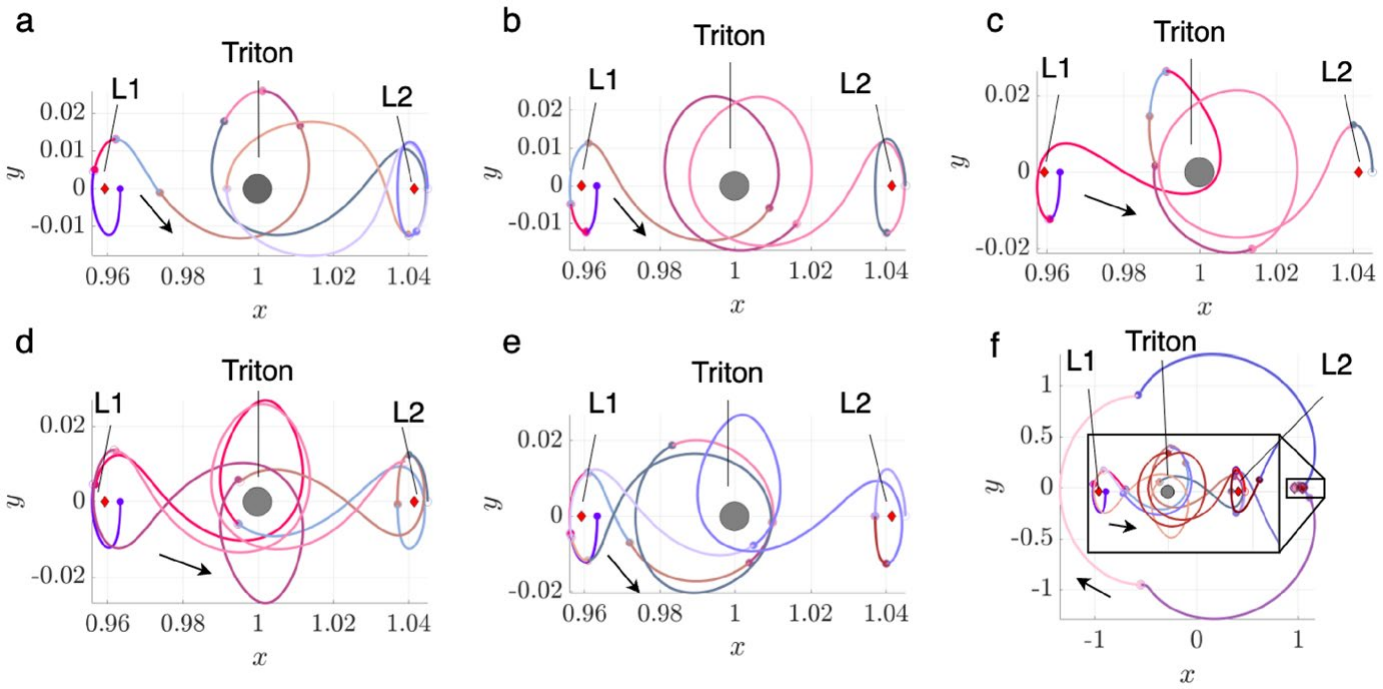
Selected examples of initial guesses for a transfer from an $L_1$ Lyapunov orbit at $C_J = 3.01400$ to an $L_2$ Lyapunov orbit at $C_J = 3.01377$ in the CR3BP

The *k*-best paths search algorithm described in Step 3 is used to generate 50 motion primitive sequences that are refined to produce geometrically diverse initial guesses between the selected $L_1$ and $L_2$ Lyapunov orbits. Generating 50 motion primitive sequences requires 163 s, whereas generating their associated initial guesses requires an additional 22 s. A subset of six of these initial guesses is presented in Fig. 7. The transfers in Fig. 7a–e complete two or more revolutions of distinct geometries around Triton, with some even revolving near $L_1$ or $L_2$ temporarily. Figure 7f displays a transfer that temporarily departs to the exterior region before returning through the $L_2$ gateway, generated from a larger graph that includes additional primitives extending into the exterior region.

### Step 5: Trajectory Correction and Optimization

Each initial guess is corrected and optimized via collocation with impulsive maneuvers. A continuous trajectory is first generated in the CR3BP using a specified maneuver placement strategy. This solution is then used as an initial guess to generate a continuous trajectory in a point mass ephemeris model of the Neptunian system. This step of the process follows the approach originally presented by Smith and Bosanac [25], with the addition of including path constraints.

#### Numerically Correcting Trajectories

Collocation is used to correct an initial guess generated in Step 4 to produce a continuous trajectory with impulsive maneuvers. This particular corrections approach relies on fitting polynomials of *N*th order to states distributed along a sequence of arcs [55]. These polynomials are constrained to 1) be continuous between arcs and 2) closely approximate solutions to a specified dynamical model. In this paper, the formulation of the collocation problem follows the approach presented by Grebow and Pavlak [56]. For brevity, this subsection provides a brief definition of each component of the collocation problem in the context of correcting trajectories in the CR3BP, using states defined in the rotating frame. However, the same formulation is straightforwardly extended to the ephemeris model by 1) using $\boldsymbol{X}_{N,sc}$ rather than $\boldsymbol{x}$, 2) adding an epoch continuity constraint, and 3) modifying the equations of motion, as presented by Smith [57].

The first step of collocation involves creating a mesh of nodes. Each initial guess is discretized into *m* segments, where the *i*th segment is split into $n_i$ arcs. The trajectory designer defines the initial number and spacing of these segments and arcs, along with the locations of impulsive maneuvers between segments or arcs. A seventh-order polynomial collocation scheme is used to place $p=7$ nodes along each arc [56]. Within this mesh, the state at the *k*th node of the *j*th arc along the *i*th segment of the trajectory is described by the state vector $\boldsymbol{x}^{i}_{j,k}$ in the rotating frame. In addition, $\Delta t^i_{j}$ is the integration time along the *j*th arc. At state $\boldsymbol{x}^{i}_{j,k}$ and time $t^{i}_{j,k}$ along the *j*th arc of the *i*th segment, the normalized time is calculated as

11 $$\begin{aligned} \tau ^{i}_{j,k} = 2((t^{i}_{j,k} - t^{i}_{j,1})/\Delta t^i_{j})- 1 \end{aligned}$$

and spans the range $[-1,1]$ along the arc. Legendre-Gauss-Lobatto (LGL) node spacing is used to place the $p=7$ nodes along each arc at normalized times that are equal to the roots of the derivative of the $(p-1)$th order Legendre polynomial [56, 58].

A free variable vector encodes the states and integration times at the odd-numbered nodes along each arc that are labeled as free nodes and used to fit each polynomial. The free variable vector $\boldsymbol{V}_{c,i}$ for the *i*th segment is expressed as

12 $$\begin{aligned} \boldsymbol{V}_{c,i} = \begin{bmatrix} \begin{bmatrix} \boldsymbol{x}_{1,1}^i \\ \boldsymbol{x}_{1,3}^i \\ \boldsymbol{x}_{1,5}^i \end{bmatrix}^T &  \begin{bmatrix}\boldsymbol{x}_{2,1}^i \\ \boldsymbol{x}_{2,3}^i \\ \boldsymbol{x}_{2,5}^i \end{bmatrix}^T &  \ldots &  \begin{bmatrix} \boldsymbol{x}_{n_i-1,1}^i \\ \boldsymbol{x}_{n_i-1,3}^i \\ \boldsymbol{x}_{n_i-1,5}^i \end{bmatrix}^T &  \begin{bmatrix} \boldsymbol{x}_{n_i,1}^i \\ \boldsymbol{x}_{n_i,3}^i \\ \boldsymbol{x}_{n_i,5}^i \\ \boldsymbol{x}_{n_i,7}^i \end{bmatrix}^T &  \begin{bmatrix} \Delta t_{1}^i \\ \Delta t_{2}^i \\ \vdots \\ \Delta t_{n_i}^i \end{bmatrix}&\end{bmatrix} \end{aligned}$$

Note that $\boldsymbol{x}^{i}_{j,7}$ is not included in $\boldsymbol{V}_{c,i}$ because the last node of the *j*th arc coincides with the first node of the $(j+1)$th arc. The free variable vector for the entire trajectory $\boldsymbol{V}$ is then defined as

13 $$\begin{aligned} \boldsymbol{V} = [\boldsymbol{V}_{c,1}, \boldsymbol{V}_{c,2}, ..., \boldsymbol{V}_{c,m}] ^{T} \end{aligned}$$

resulting in a total of $((3p-2)\sum _{i=1}^{m}n_i +6m)$ free variables.

A constraint vector is defined to enforce continuity between segments and the dynamics at the even-numbered nodes along each arc that are labeled as defect nodes. The continuity constraint vector between the *i*th and $(i+1)$th segments is defined as

14 $$\begin{aligned} \boldsymbol{F}_c^i = \left\{ \begin{array}{lcl} (\boldsymbol{x}_{1,1}^{i+1} - \boldsymbol{x}_{n_i,p}^{i})^T \ \text {if natural motion} \\ (\boldsymbol{r}_{1,1}^{i+1} - \boldsymbol{r}_{n_i,n}^{i})^T \ \text {if impulsive maneuver applied} \end{array} \right. \end{aligned}$$

for $i<m$. Next, the free nodes are used to fit a polynomial $\boldsymbol{q}(\boldsymbol{x})$ in the rotating frame along each arc. These polynomials are compared to the system dynamics at the defect nodes to define the following defect constraint vectoralong the *j*th arc of the *i*th segment:

15 $$\begin{aligned} \boldsymbol{F}_{d,j}^i = \begin{bmatrix} (\dot{\boldsymbol{q}}_{j,2}^i(\tau _2) - \dot{\boldsymbol{x}}_{j,2}^i)w_2 \\ (\dot{\boldsymbol{q}}_{j,4}^i(\tau _4) - \dot{\boldsymbol{x}}_{j,4}^i)w_4 \\ (\dot{\boldsymbol{q}}_{j,6}^i(\tau _{6}) - \dot{\boldsymbol{x}}_{j,6}^i)w_{6} \end{bmatrix}^T \end{aligned}$$

where $w_k$ is the LGL weight associated with the *k*th collocation node and $\dot{\boldsymbol{q}}$ is the derivative of the polynomial along the arc with respect to normalized time $\tau$. In this expression, $\dot{\boldsymbol{x}}$ is the normalized time derivative of the state vector $\boldsymbol{x}_{j,k}^i$ calculated as

16 $$\begin{aligned} \dot{\boldsymbol{x}}_{j,k}^i = \frac{\Delta t_j^i}{2}\boldsymbol{g}(\boldsymbol{x}_{j,k}^i) \end{aligned}$$

where $\boldsymbol{g} = [\dot{x},\dot{y},\dot{z}, \ddot{x}, \ddot{y}, \ddot{z}]$. For all $n_i$ arcs along the *i*th segment, the defect constraint vector is

17 $$\begin{aligned} \boldsymbol{F}_d^i = \begin{bmatrix} \boldsymbol{F}_{d,1}^i, \boldsymbol{F}_{d,2}^i, \ldots , \boldsymbol{F}_{d,n_i}^i \end{bmatrix} \end{aligned}$$

Finally, the constraint vector $\boldsymbol{F(V)}$ for the entire trajectory is defined as

18 $$\begin{aligned} \boldsymbol{F(V)} = \begin{bmatrix} \boldsymbol{F}_{c}^1, \boldsymbol{F}_{c}^2, \ldots , \boldsymbol{F}_{c}^{m-1}, \boldsymbol{F}_{d}^1, \boldsymbol{F}_{d}^2, \ldots , \boldsymbol{F}_{d}^m \end{bmatrix}^T \end{aligned}$$

For a continuous transfer to exist, this constraint vector must equal zero within a tolerance of $10^{-12}$ in the Neptune-Triton CR3BP and $10^{-9}$ in the ephemeris model.

Where appropriate, additional path constraints are also enforced. In this paper, an inequality constraint is defined to ensure that the minimum distance from body *i* at node *j*, $d_{i,j}$, does not fall below a specified lower bound, $d_{i, min}$, i.e., $d_{i,j} \ge d_{i, min}$. Additional constraints on the flight time and single or total maneuver magnitude could be imposed as desired. In addition, equality constraints are used to enforce that the initial and final states along the transfer depart from the initial orbit and arrive at the final orbit, respectively. Future work will incorporate additional constraints to ensure that the spacecraft does not pass through the rings of Neptune.

Once a corrected transfer has been recovered, the mesh is refined to improve the accuracy of the polynomial approximation of the trajectory. This refinement step enables arcs to possess different lengths in more or less sensitive regions of the phase space. The mesh refinement step aims to equally distribute the error on the constraint nodes along the arcs of the solution [56]. In this paper, hybrid mesh refinement is implemented using the approach presented by Grebow and Pavlak and the method for error redistribution by Carl de Boor [56, 59, 60].

#### Constrained Local Optimization

Collocation is combined with multi-objective optimization and continuation to generate a family of transfers that balance resembling the initial guess while reducing the maneuver requirements [25]. First, the free variables of the optimization problem correspond to the vector $\boldsymbol{V}$ defined in Eq. (13). A multi-objective cost function *J* is then defined as follows to balance these two competing objectives:

19 $$\begin{aligned} J(\boldsymbol{V}) = w_{geo}\sum _{i=1}^{n_n}(\Delta \boldsymbol{r}_{i,ig-ct}^2) + w_{man}\sum _{j=1}^{n_m}(\Delta v_j^2) \end{aligned}$$

where $\Delta \boldsymbol{r}_{i,ig-ct}$ is themagnitude of the difference between the position vector of the *i*th of $n_n$ collocation nodes along the initial guess and the current mesh, $\Delta v_j$ is the magnitude of the *j*th of $n_m$ impulsive maneuvers, and $w_{geo}$ and $w_{man}$ are user-defined scaling factors [25]. Note that the second term of Eq. (19) minimizes the control energy, rather than the total maneuver magnitude, to facilitate convergence [61]. The constraints of the optimization problem are defined by the collocation constraint vector $\boldsymbol{F}(\boldsymbol{V})$ in Eq. (18), along with any selected path constraints. The gradients of the constraints and objective function are calculated analytically. For a selected combination of $w_{geo}$ and $w_{man}$, constrained local optimization is implemented using sequential quadratic programming in MATLAB via the fmincon function with an optimality tolerance of $10^{-3}$ [50].

Continuation is used to gradually adjust the values of $w_{geo}$ and $w_{man}$ to generate a family of solutions to the multi-objective optimization problem [25]. At the first iteration, the collocation-based optimization problem is solved with $w_{geo} = 0.99$ and $w_{man} = 0.01$ to generate a continuous and feasible transfer that closely resembles the primitive-based initial guess. After this first iteration, the mesh is also refined as described in the previous subsubsection. Then, ten continuation steps are used to gradually adjust the weights of the multi-objective function in equal increments to $w_{geo} = 0.01$ and $w_{man} = 0.99$. Note that $w_{geo} = 0$ and $w_{man} = 1$ are not used as the final values as fmincon sometimes struggles to return a nearby solution within a reasonable computational time; the use of alternative optimization implementations is an area of future work. At each continuation step, the last known solution supplies the initial guess, and the optimization problem is solved again. However, if the magnitude of any maneuver falls below 0.01 m/s, the maneuver is removed and the trajectory is corrected again. As a result of this continuation approach, the transfer obtained at each step gradually evolves away from the initial guess as the maneuver requirements decrease. At the end of this continuation procedure, the trajectory that minimizes Eq. (19) with $w_{geo} = 0.01$ and $w_{man} = 0.99$ is labeled as an optimal trajectory as it possesses the lowest maneuver requirements within a family of solutions. Future work will also include minimizing the total maneuver magnitude, rather than the maneuver magnitude squared, upon completion of the continuation process.

#### Examples of Continuous Transfers

**Fig. 8:**
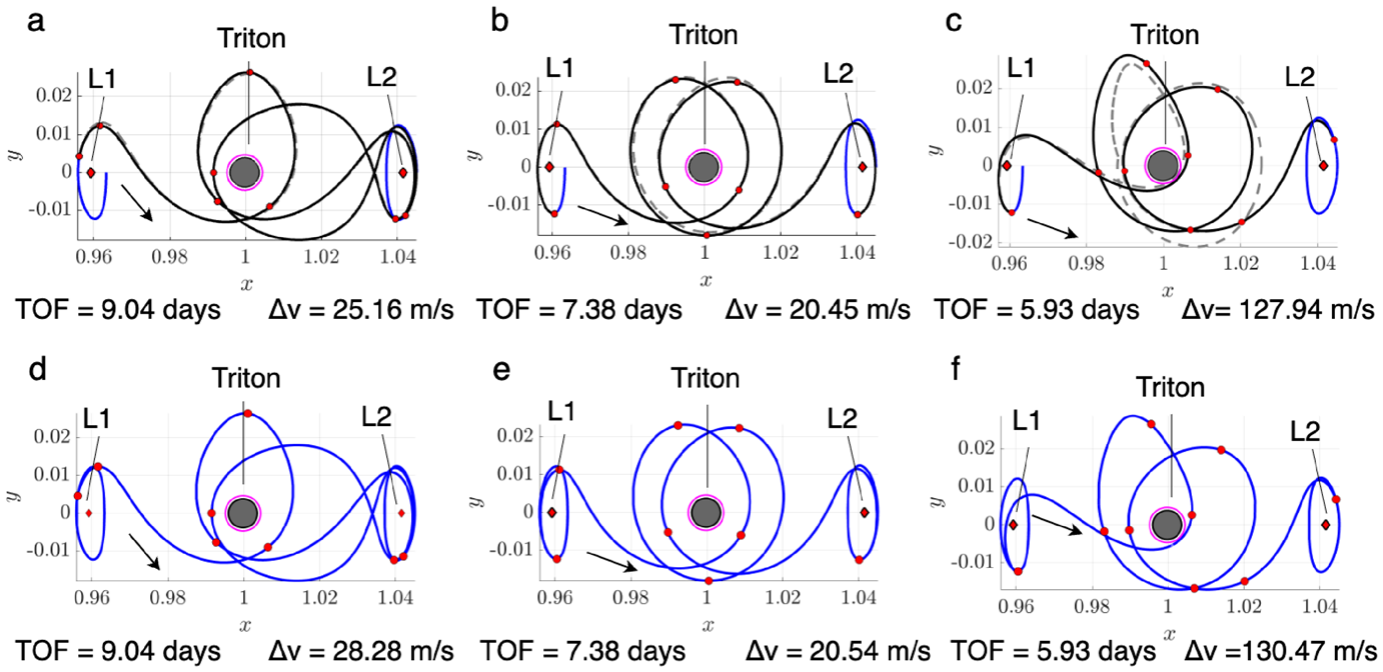
Selected optimal trajectories, with with $w_{geo} = 0.01$ and $w_{man} = 0.99$, for a transfer from an $L_1$ Lyapunov orbit at $C_J = 3.01400$ to an $L_2$ Lyapunov orbit at $C_J = 3.01377$ in the **a**–**c** CR3BP and **d**–**f** ephemeris model

The corrections and optimization procedure is demonstrated for the example used throughout this section to design a transfer from an $L_1$ Lyapunov orbit at $C_J = 3.01400$ to an $L_2$ Lyapunov orbit at $C_J = 3.01377$. The first three initial guesses from Fig. 7 are corrected and optimized in the CR3BP with a constraint on the minimum distance from Triton corresponding to an altitude of $h = 300$ km. For each transfer, impulsive maneuvers are placed at the locations of maximum curvature along the initial guess, as well as at departurefromand arrival at the periodic orbits; using and analyzing alternative maneuver placement strategies is a suitable avenue for future work. Following the continuation-based approach to reducing the maneuver requirements, the transfers generated with $w_{geo} = 0.01$ and $w_{man} = 0.99$ are plotted in Fig. 8a–c. Each subplot displays the initial and final periodic orbits in blue, the continuous transfer in black, the initial guess in a gray dashed curve, and the maneuver locations as red dots. The magenta circle around Triton depicts the minimum altitude constraint. The flight time, defined as the time between departure from the initial orbit and arrival into the target orbit, is listed below each transfer along with the total maneuver magnitude. Figure 8a, b demonstrate that the corrected transfers retain the geometry of the primitive-based initial guesses; the noticeable shift in the path in Fig. 8c is a result of the minimum altitude constraint with respect to Triton.

The selected optimal trajectories in the Neptune-Triton CR3BP are corrected in the ephemeris model. Three additional revolutions of the initial and final orbits are added to the beginning and end of each transfer to encourage boundedness in the vicinity of the reference orbits. In addition, an initial epoch on December 1st, 2045 UTC is used in all cases. The resulting transfers are plotted in a pulsating Neptune-Triton rotating frame in Fig. 8d–f with the flight time and total maneuver magnitude listed below each transfer. These transfers closely resemble the trajectories in the Neptune-Triton CR3BP, with similar flight times and maneuver requirements.

## Results

The presented motion primitive approach to trajectory design is applied to two scenarios in the Neptunian system. Section 4.1 focuses on designing trajectories from a high-energy arrival into the Neptunian system to insertion into a science orbit. The second example, presented in Sect. 4.2, focuses on designing low-energy transfers from a Neptune-centered resonant orbit to a low prograde orbit around Triton. These two examples are selected to span distinct ranges of the Jacobi constant and, therefore, encompass different regions of the solution space; leverage distinct sets of motion primitives during graph construction; and produce planar trajectories with substantially different itineraries. The results in this section are computed using MATLAB R2022a [50] on a 2020 MacBook Pro with M1 chip and 8 GB of RAM.

Although these applications focus on generating planar trajectories, the presented approach could also be used to design spatial trajectories. In that case, spatial motion primitives would need to be generated in Step 1, substantially increasing the size of the library. Then, the motion primitive approach to trajectory design would proceed as described in Steps 2–5. However, as the total number of motion primitives increases, the computational time associated with constructing and searching the motion primitive-informed graph in Steps 2–3 will also increase. Addressing this limitation of the current approach is a focus of ongoing work.

### High-Energy Transfer Design

The high-energy trajectory design case is used to generate an array of geometrically diverse planar trajectories to insert into a Neptune-centered resonant orbit following arrival into the Neptunian system. In this example, the target orbit is selected as a 1:7 resonant orbit that passes through the orbits of Proteus and Hippocamp, two of Neptune’s moons. This target orbit can potentially support meaningful scientific observations of the inner moons of the system, allowing for relevant science to be performed immediately upon arrival in the system. Both the initial and final conditions possess low values of the Jacobi constant.

#### Graph Construction

To construct the motion primitive-informed graph, the initial condition in the Start set of the high-level graph structure is defined as an arc that occurs at the end of an interplanetary transfer from Earth and passes through the Neptunian system. This arc is generated from a state at periapsis with respect to Neptune provided by Dr. Reza Karimi from a prior study at the NASA Jet Propulsion Laboratory (R. Karimi, personal communication, July 2023). This state has the following characteristics:

- Epoch at periapsis, $t_1$: October 2, 2045, 11:52:51 UTC
- Periapsis altitude relative to Neptune: 2460.11 km
- Hyperbolic excess velocity, $v_{\infty }$: 11.5252 km/s
- The angle between the velocity vector and the XY-plane of the Neptune-fixed frame labeled ‘IAU_NEPTUNE’ in the ‘pck00011.tpc’ kernel: $\delta = 8.3778^{\circ }$

**Fig. 9:**
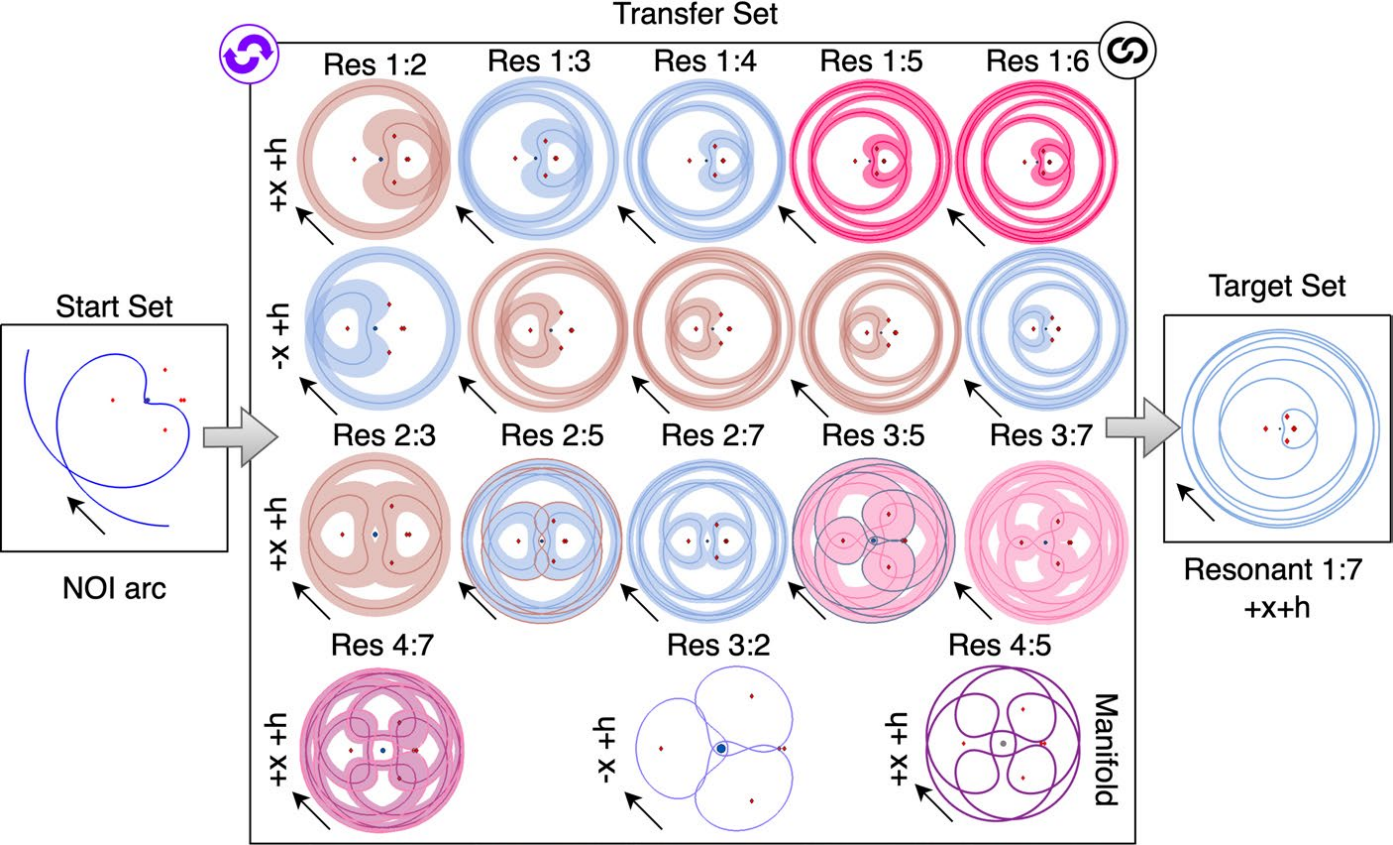
High-level structure of the motion primitive-informed graph for a transfer from interplanetary arrival to a 1:7 resonant orbit

Because the original initial condition does not lie in the Neptune-Triton plane, the state components are modified to obtain a state along a planar trajectory in the Neptune-Triton rotating frame with a similar velocity, energy, and distance [62]. This state is propagated backward and forward in time for 3.75 days in the CR3BP to generate an arc, as depicted in the Start set of Fig. 9 in the Neptune-Triton rotating frame.

The selected target orbit is a 1:7 resonant orbit with $C_J = 1.8$ and a period of 41.14 days in the Neptune-Triton CR3BP. This periodic orbit is plotted in the Target set of Fig. 9 in the Neptune-Triton rotating frame. This resonant orbit possesses a periapsis distance with respect to Neptune of $r_{pN} = 108,973.86$ km. This orbit also crosses the low-eccentricity orbits of Proteus and Hippocamp that possess semi-major axes of $a_P = 117,647$ km and $a_H = 105,300$ km, respectively [30]. These characteristics could potentially be useful for a spacecraft performing scientific observations of the Neptunian system. Incorporating the precise locations of the inner moons to design flybys, connecting target orbit selection to scientific requirements, or performing a trade study of various candidate science orbits are interesting avenues for future work.

The Transfer set of the high-level graph structure is selected to encompass motion primitives with similar geometry and Jacobi constant to the initial and target orbits. The middle box of Fig. 9 depicts the selected primitives and their region of existence. In this box, each subfigure displays selected primitives from a single family: *p*:*q* resonant orbits are labeled as “Res p:q” above each column or individual subfigure, the annotation $\pm x \pm h$ to the left of each row or individual subfigure indicates the location of the initial apse and direction of motion as defined in Step 1, and the label “Manifold” on the right of any subfigures indicates that the motion primitives summarize arcs along the stable and unstable manifolds of the depicted periodic orbit. The purple symbol at the top left corner of the Transfer set indicates that distinct motion primitives generated from a single orbit family or manifold can be connected. Similarly, the black symbol at the top right corner of the Transfer set indicates that motion primitives generated from distinct orbit families or manifolds in the set can be connected. The motion primitives within the Transfer set are selected for this example because 1) the associated values of the Jacobi constant lie between the arcs in the initial and target sets and 2) they tend to capture the array of observed geometries across a broader set of motion primitives summarizing resonant orbits and their stable and unstable manifolds at these energy levels.

The high-level graph structure, composed of 266 motion primitives, is used to form a motion primitive-informed graph that is composed of 3871 nodes and 255,550 edges. The initial state, integration time, and Jacobi constant of all primitives included in this graph are provided as supplementary data in the form of a text file (Online Resource 3). On the specified computer, this final graph is generated in 29.33 min. A total of 50 motion primitive sequences are obtained from the graph in 59.08 min. Initial guesses are then generated from these sequences in 32.93 min.

#### Trajectory Tradespace

A subset of 16 initial guesses is corrected and optimized in the CR3BP. At this step, the altitude relative to Neptune is constrained to not fall below 10 km. However, larger minimum altitude thresholds could be used in future work to support planetary protection or incorporate precise mission requirements. In addition, similar to the technical approach, impulsive maneuvers are placed at the locations of maximum curvature along the initial guess and departure from and insertion into the boundary arcs; adjusting the locations of these maneuvers and exploring their influence on the transfers is another interesting avenue for future work.

**Fig. 10:**
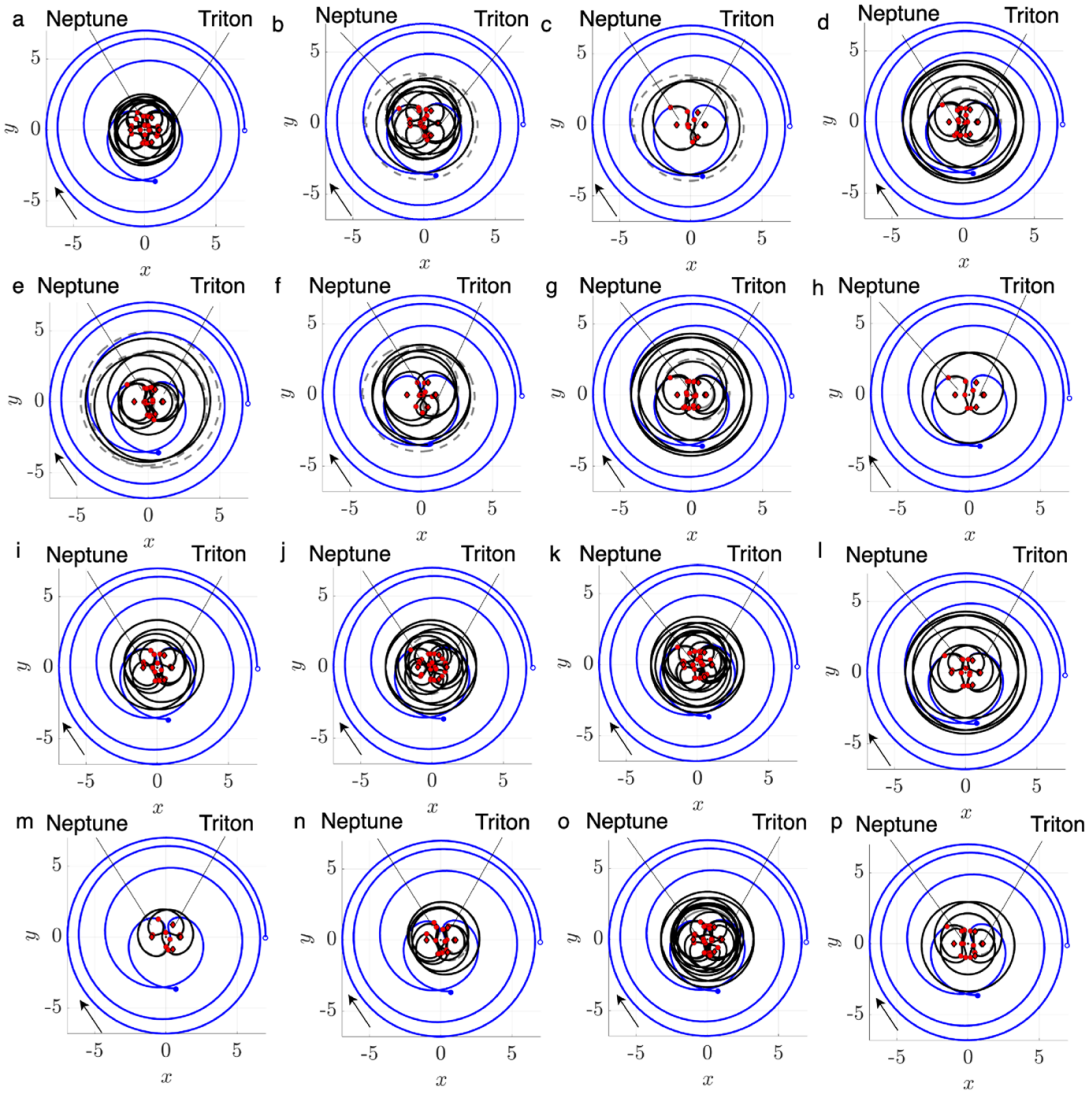
Optimal trajectories, with $w_{geo} = 0.01$ and $w_{man} = 0.99$, from interplanetary arrival to a 1:7 resonant orbit in the Neptune-Triton CR3BP, displayed in the Neptune-Triton rotating frame

The optimal transfers that minimize Eq. (19) with $w_{geo} = 0.01$ and $w_{man} = 0.99$ are presented in the Neptune-Triton rotating frame in Fig. 10.

In each plot, the initial interplanetary arrival arc and the target 1:7 resonant orbit are displayed in blue, the transfer is plotted in black, and the initial guess is displayed as a gray dashed curve. The locations of impulsive maneuvers are indicated by red circles, whereas filled and empty blue circles locate the initial and final states of the transfer, respectively. The direction of motion is also indicated by a black arrow. For the transfer in each subfigure of Fig. 10, the associated time of flight and total maneuver magnitude are listed in Table 1.

**Table 1:** Time of flight and total $\Delta v$ for optimal transfers with $w_{geo} = 0.01$ and $w_{man} = 0.99$ in Fig. 10

Transfer	a	b	c	d	e	f	g	h
TOF (days)	80.25	57.82	16.77	57.82	51.53	39.58	57.40	16.45
$\Delta v_{tot}$ (km/s)	4.26	2.76	1.69	3.73	4.75	4.72	3.34	1.76

Transfer	i	j	k	l	m	n	o	p
TOF (days)	51.07	75.06	92.76	57.35	15.85	45.56	104.69	34.43
$\Delta v_{tot}$ (km/s)	3.47	4.39	4.25	2.76	2.53	3.93	3.64	3.28

The generated trajectories possess distinct geometries while closely resembling their initial guesses. Within each individual subfigure, the corrected transfer tends to either remain close to the gray dashed curve associated with the initial guess or shift closer to Neptune while retaining a similar shape. Across the different subfigures, the transfers possess distinct geometries as they complete a distinct number of revolutions and visit different regions of the Neptunian system. These two observations demonstrate that the motion primitive approach supports automatically generating a diverse array of transfers to reach a specified target science orbit while satisfying path constraints.

**Fig. 11:**
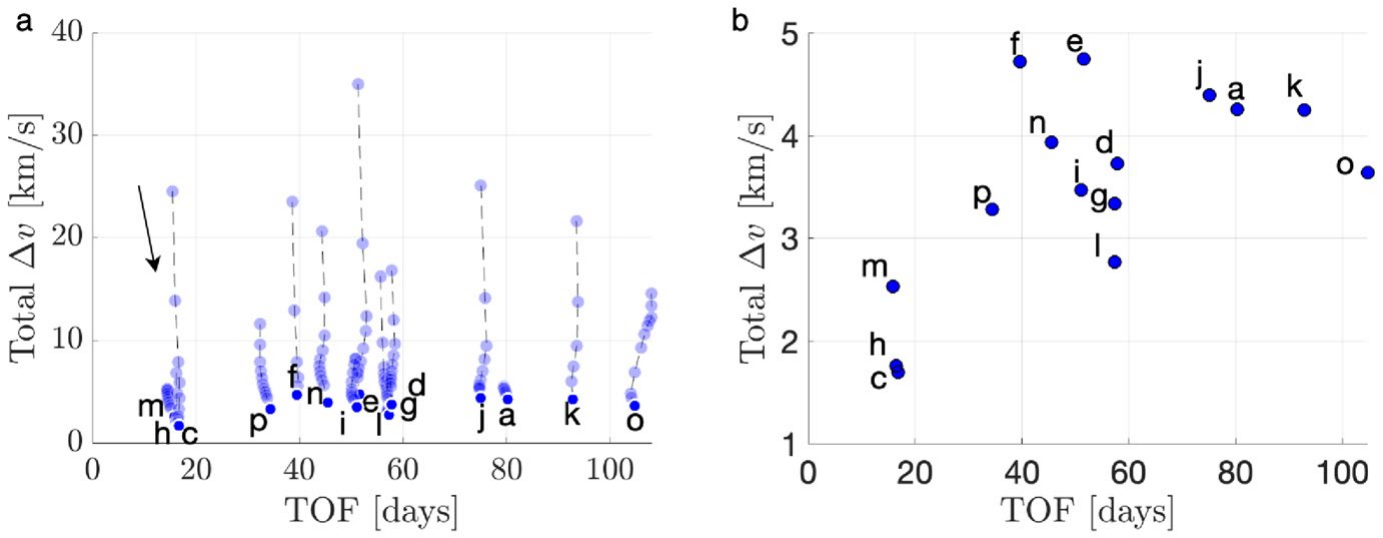
Flight time and total $\Delta v$ for **a** each family generated during continuation to resemble the transfers in Fig. 10 and **b** the optimal transfers with $w_{geo} = 0.01$ and $w_{man} = 0.99$ in Fig. 10

These geometrically distinct trajectories are useful for studying a segment of the trajectory tradespace. Figure 11a depicts the flight time and total $\Delta v$ for the family of transfers that resembles each subfigure in Fig. 10 while varying the values of $w_{geo}$ and $w_{man}$.

Then, Fig. 11b plots these two characteristics for only the transfers generated with $w_{geo} = 0.01$ and $w_{man} = 0.99$ at the end of the continuation process. In each case, the associated transfers are labeled consistent with Fig. 10. In Fig. 11a, the total maneuver requirement decreases substantially with small changes to the total time of flight across each family: in most but not all cases, the flight time increases when the $\Delta v$ decreases. In Fig. 11b, the solutions with the lowest maneuver requirements in each family exhibit an interesting trend across the tradespace: the transfers with the lowest flight time tend to also possess the lowest total maneuver requirements. In fact, transfers c and h possess the lowest total $\Delta v$ values of 1.69–1.76 km/s, flight times under 17 days, and paths that geometrically resemble a 2:7 resonant orbit. Further exploring these trends, analyzing whether alternative maneuver placement could further reduce these maneuver requirements, and comparing these characteristics to mission requirements are interesting avenues of future work.

**Fig. 12:**
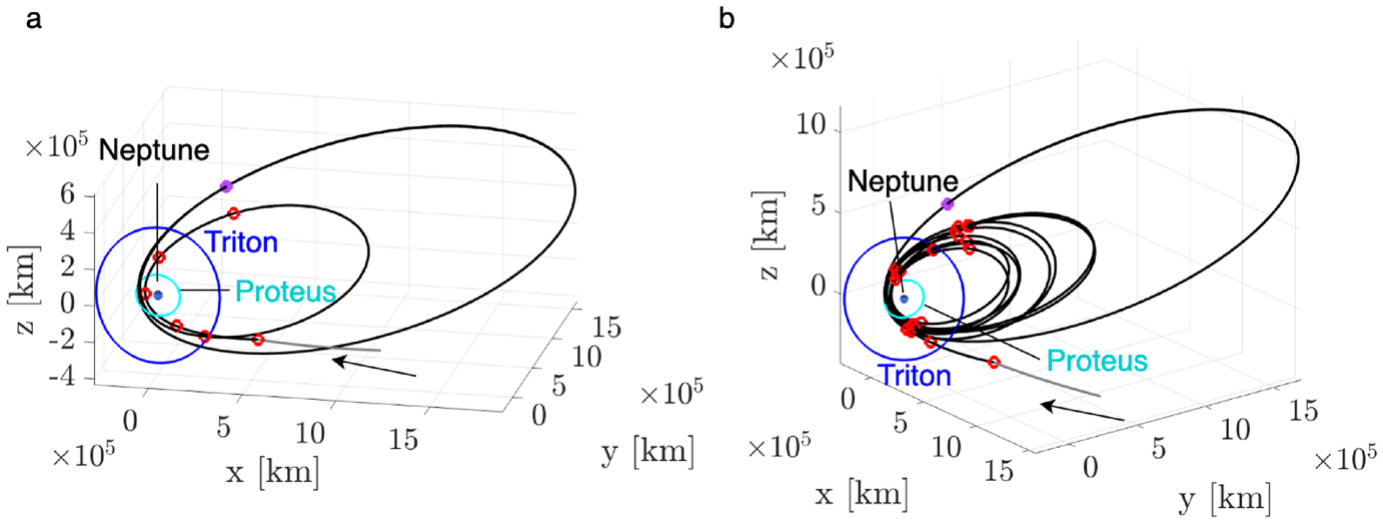
Two trajectories from interplanetary arrival to a 1:7 resonant orbit corrected in an ephemeris model of the Neptunian system, displayed in the Neptune-centered ICRF with **a** $\Delta v = 1.97$ km/s and $TOF = 16.77$ days and **b** $\Delta v = 5.69$ km/s and $TOF = 75.06$ days

Two transfers are corrected in an ephemeris model and visualized in the inertial frame. Figure 12 depicts these two transfers corrected in the ephemeris model to resemble the transfers in Fig. 10c, j. The initial guess used to generate each transfer is the optimal solution with $w_{geo} = 0.01$ and $w_{man} = 0.99$ from the Neptune-Triton CR3BP. In these figures, the initial arrival arc is depicted in gray, the first maneuver in magenta, and the rest of the transfer in black. The red circles locate impulsive maneuvers, and the purple circle locates the insertion into the target resonant orbit. The final trajectory after the last maneuver is colored gray. For reference, the orbits of Triton and Proteus are plotted in blue and cyan, respectively. Although not displayed here, these transfers that exist in the ephemeris model closely resemble the paths corrected in the Neptune-Triton CR3BP when visualized in the rotating frame. Furthermore, they possess a similar flight time and total $\Delta v$ to the transfers across the two dynamical models. This example demonstrates that the use of motion primitives, generated in a low-fidelity dynamical model, supports the generation of geometrically distinct transfers even in an ephemeris model of the Neptunian system, as long as close flybys of other bodies do not occur. In this case, the characteristics of trajectories generated in the CR3BP supply a close estimate of the associated characteristics in the ephemeris model.

### Low-Energy TransferDesign

Low-energy trajectories are generated from a 3:2 resonant orbit to a low prograde orbit around Triton. Trajectories orbiting planetary moons can provide strategic locations for longer-term scientific observations or deployments of probes. However, compared to the previous example, the trajectory design space is substantially more complex at the higher values of the Jacobi constant associated with the selected boundary conditions, which also revolve around distinct celestial bodies in the Neptunian system.

#### Graph Construction

**Fig. 13:**
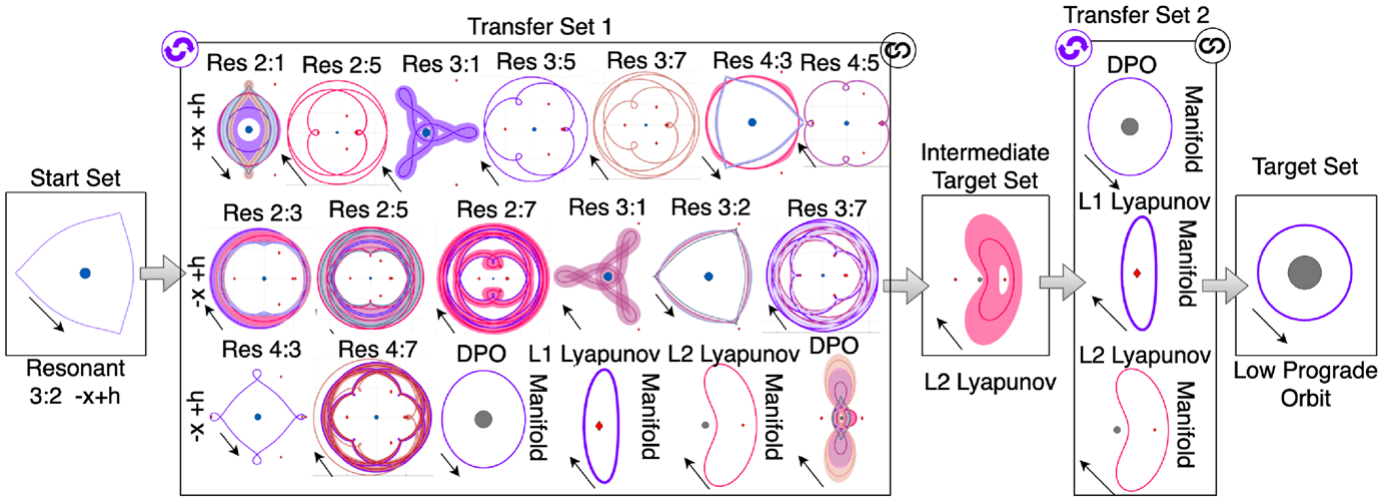
High-level structure of the motion primitive-informed graph for a transfer from a 3:2 resonant orbit to a low prograde orbit about Triton

To define the high-level graph structure, the Start set consists of a 3:2 resonant orbit that is prograde in the rotating frame with a semi-major axis of $a = 407,885.96$ km relative to Neptune. In the Neptune-Triton rotating frame and the CR3BP, this orbit possesses a Jacobi constant of $C_J = 3.02884$, a period of 11.74 days, and no planar unstable modes. This orbit is plotted in the leftmost box of Fig. 13. Depending on instrument capabilities, this orbit could support observing Neptune from Triton’s orbital plane, which is inclined by $157.345^\circ$ with respect to Neptune’s equator.

The Target set consists of a low prograde orbit at $C_J = 3.0216$ with an orbital period of 11.850 h. With a minimum altitude of approximately 2400 km, this low prograde orbit could support close observation of Triton while also limiting the impact of higher-order gravitational perturbations that have not yet been mapped. Furthermore, at $C_J = 3.0216$, the $L_3$ gateway is open. Accordingly, a spacecraft could traverse any region within the system before or after reaching this science orbit. However, in this case, an insertion or departure maneuver would be required as this orbit is stable in the *xy*-plane. This orbit is plotted in the rightmost box of Fig. 13.

To demonstrate the use of a desired itinerary in the design of a high-level graph structure, three blocks are added between the boundary conditions. First, a waypoint is introduced in the form of an $L_2$ Lyapunov orbit with $C_J= 3.0037$ and is labeled as the “Intermediate Target Set” located in the third block of Fig. 13. This waypoint may be useful for collecting observations or data prior to approaching Triton. Furthermore, this orbit possesses stable and unstable manifolds that traverse the entire system, and are useful in guiding the trajectory from the vicinity of Neptune or the exterior region to the $L_2$ gateway and then to the vicinity of Triton. Accordingly, “Transfer Set 1”, as depicted in Fig. 13, contains primitives summarizing 1) various resonant orbits as well as 2) periodic orbits in the vicinity of Triton and, when they exist, their stable and unstable manifolds. Finally, “Transfer Set 2” is depicted in Fig. 13 and is composed of motion primitives that summarizearcs along stable and unstable manifolds of distant prograde, $L_1$ Lyapunov, and $L_2$ Lyapunov orbits. These motion primitives in “Transfer Set 2” are also constrained to 1) remain within a distance of 0.1 nondimensional units from Triton, and 2) not pass within 10 km from Triton’s surface. These constraints ensure that the spacecraft directly approaches the target orbit from the intermediate waypoint. In each transfer set, the orbits and their manifolds possess Jacobi constants close to the range of values spanned by the boundary conditions.

The high-level graph structure, composed of 1092 primitives, is used to form a motion primitive-informed graph that is composed of 10,126 nodes and 838,869 edges. The initial state, integration time, and Jacobi constant of all primitives in this graph are provided as supplementary data in Online Resource 4. This graph is generated in 3.13 hours, and searching the graph for 50 initial guesses requires approximately 2 days. The 50 motion primitive sequences are then refined to produce initial guesses in 6 h. These long runtimes are due to the graph size, longer primitive sequences, and the use of serial computations; ongoing work to substantially reduce this computational time includes improving code efficiency and the use of parallel computing, reformulating the motion-primitive graph, and using alternative search algorithms.

#### Trajectory Tradespace

**Fig. 14:**
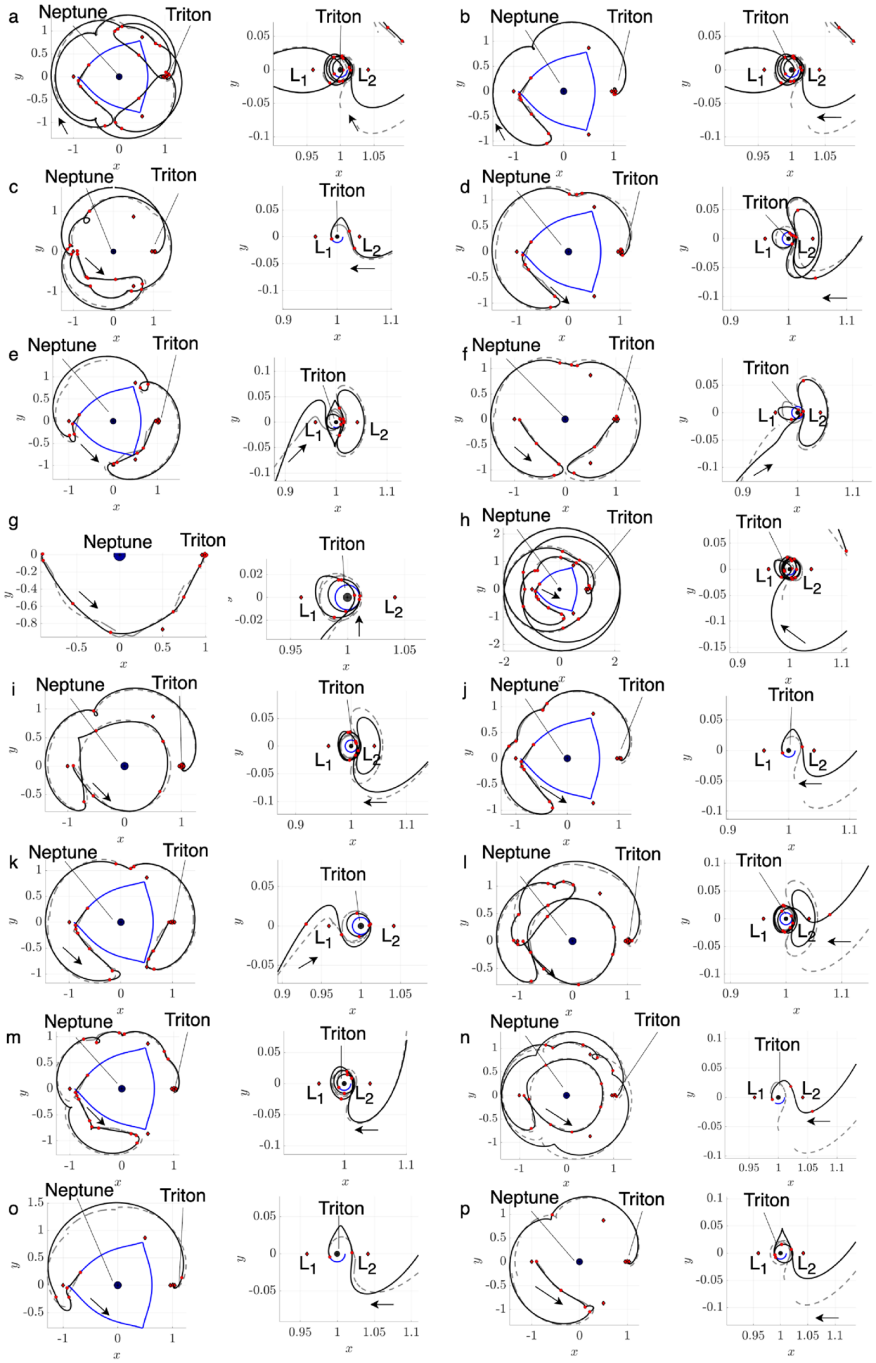
Optimal trajectories with $w_{geo} = 0.01$ and $w_{man} = 0.99$ from a 3:2 resonant orbit to a low prograde orbit about Triton in the Neptune-Triton CR3BP, displayed in the Neptune-Triton rotating frame

**Fig. 15:**
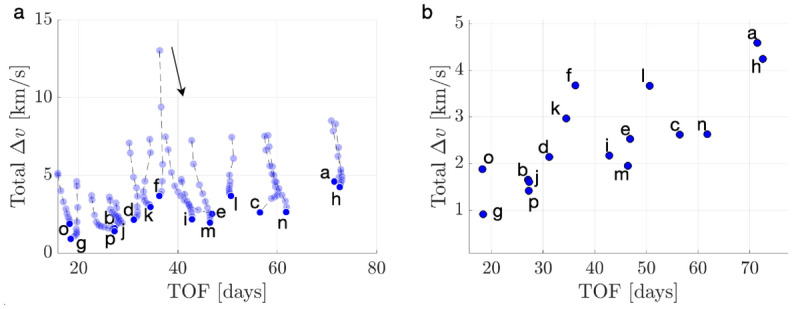
Flight time and total $\Delta v$ for **a** each family generated during continuation to resemble the transfers in Fig. 14 and **b** the optimal transfers with $w_{geo} = 0.01$ and $w_{man} = 0.99$ in Fig. 14

Selected initial guesses are corrected and optimized in the CR3BP. In this example, the altitude relative to Triton is constrained to not fall below 10 km. The resulting transfers, which minimize Eq. (19) with $w_{geo} = 0.01$ and $w_{man} = 0.99$, are presented in the Neptune-Triton rotating frame in Fig. 14. This figure uses the same configuration as Fig. 11. However, within each subfigure, each transfer is accompanied to the right by a zoomed-in view of the vicinity of Triton. In addition, the time of flight and total $\Delta v$ associated with each trajectory are listed in Table 2 with a graphical representation of the tradespace in Fig. 15.

**Table 2:** Time of flight and total $\Delta v$ for optimal transfers with $w_{geo} = 0.01$ and $w_{man} = 0.99$ in Fig. 14

Initial Guess	a	b	c	d	e	f	g	h
TOF (days)	71.48	27.09	56.50	31.19	46.88	36.22	18.42	72.57
$\Delta v_{tot}$ (km/s)	4.58	1.65	2.62	2.14	2.52	3.67	0.91	4.24

Initial Guess	i	j	k	l	m	n	o	p
TOF (days)	42.85	27.28	34.45	50.65	46.47	61.79	18.22	27.22
$\Delta v_{tot}$ (km/s)	2.17	1.60	2.96	3.66	1.95	2.64	1.88	1.42

The trajectories in Fig. 14 exhibit geometric diversity across the set before reaching the $L_2$ gateway. The majority of trajectories depart the initial orbit and immediately complete a partial revolution around $L_3$ to change the direction of motion from prograde to retrograde relative to Neptune. Then, their paths either complete a distinct number of revolutions in the rotating frame or possess a distinct shape before reaching the $L_2$ gateway. However, trajectory g in Fig. 14 is the only transfer that follows a consistently prograde path towards the $L_2$ gateway. Trajectory g also requires the lowest total $\Delta v = 0.91$ km/s and the second lowest flight time of 18.42 days, potentially indicating that a change in direction of motion might increase the overall maneuver requirement. Finally, in each case, the optimal path tends to remain close to the gray dashed line associated with the initial guess.

Upon reaching the vicinity of Triton, the optimal solution tends to shift noticeably from the initial guess while still retaining the general geometry. For the transfers plotted in Fig. 14a, j, n, this shift is expected as continuity is enforced. The large discontinuities in these initial guesses are predominantly due to the use of an average neighborhood size when assessing sequential composability between motion primitives that remain close to Triton with smaller regions of existence and motion primitives that traverse the system with large regions of existence; addressing this limitation is a focus of ongoing work. Some transfers, such as those depicted in Fig. 14c, e, o, noticeably evolve during continuation to reduce the maneuver requirements: as the apoapsis distance increases and its speed decreases, the shape of the associated segments of the path evolves to resemble a DPO. The use of distance-based scaling factors in the objective function in Eq. (19) may support maintaining geometric resemblance to the initial guess when the path visits regions of distinct sensitivity; such analysis would be an interesting avenue for future work. Finally, the trajectories in Fig. 14e–g, k approach Triton through the $L_1$ gateway with transfers g and k using only a small segment of the $L_2$ Lyapunov orbit waypoint in their initial guesses.

The flight time and total $\Delta v$ tradespace is depicted in Fig. 15. Similar to the previous example, Fig. 15a displays the flight time and total $\Delta v$ tradespace for each solution geometry as the values of $w_{geo}$ and $w_{man}$ evolve during continuation. Furthermore, in Fig. 15b, the transfers with the lowest flight times tend to possess lower maneuver requirements for the select maneuver placement strategy.

**Fig. 16:**
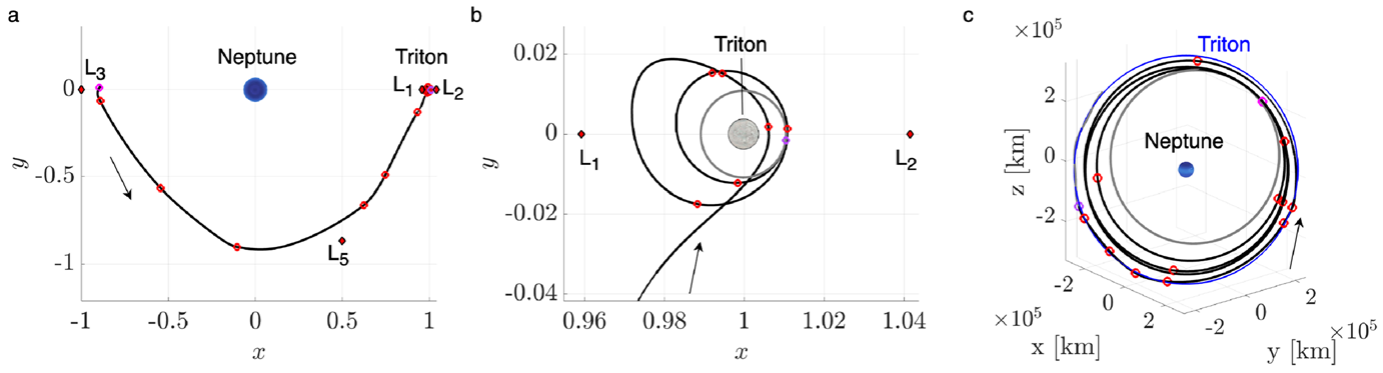
Trajectory in Fig. 14 g corrected in an ephemeris model of the Neptunian system with a total $$\Delta v = 0.9115$$ km/s and TOF $$ = 18.42$$ days, displayed **a** in the Neptune-Triton rotating frame with a global view of the trajectory around Neptune, **b** in the Neptune-Triton rotating frame with a zoomed-in view in the vicinity of Triton, and **c** in the ICRF centered on Neptune

The trajectory with the lowest total $\Delta v$, i.e., trajectory g in Fig. 14, is corrected in an ephemeris model. The corrected trajectory is plotted in the Neptune-Triton rotating frame in Fig. 16a, with a zoomed-in view of the vicinity of Triton in Fig. 16b, and in the Neptune-centered ICRF in Fig. 16c. Fig. 16a, b demonstrate that the geometry of the trajectory designed in the CR3BP is maintained in the ephemeris model. Furthermore, Fig. 16c demonstrates that the spacecraft departs from the Neptune-centered resonant orbit after the maneuver in magenta, and travels towards the orbit of Triton in blue by slowly increasing its distance from Neptune until inserting into the Triton-centered orbit with the maneuver colored purple.

## Conclusions

This paper presents an updated motion primitive approach to spacecraft trajectory design in multi-body systems. First, motion primitives are generated in the Neptune-Triton CR3BP by grouping periodic orbits according to their curvature time history and clustering arcs along stable and unstable invariant manifolds by their geometry. Each motion primitive summarizes arcs with a similar geometry that span its region of existence. Then, a new motion primitive-informed graph is defined to 1) reflect the sequential composability of pairs of primitives, thereby improving the quality of initial guesses for complex trajectories, and 2) incorporate additional path constraints to support automatically generating constrained trajectories. Next, a *k*-best paths graph search algorithm is presented to generate motion primitive sequences that are diverse and can span various lengths. A new graph-based approach is then used to generate a globally optimal initial guess from each sequence of primitives and their associated regions of existence. Each initial guess is corrected using collocation and multi-objective optimization to produce a constrained, continuous trajectory in the CR3BP and then in an ephemeris model.

The updated motion primitive approach is applied to two planar scenarios in the Neptune-Triton system to generate a tradespace of trajectories. First, 16 high-energy transfers are designed from arrival into the Neptunian system to a 1:7 resonant orbit. These geometrically diverse transfers span flight times of 15.85$-$104.69 days and a total $\Delta v$ of 1.69$-$4.75 km/s with impulsive maneuvers near the locations of maximum curvature in the rotating frameand at the boundary conditions. Next, 16 low-energy transfers are designed from a Neptune-centered 3:2 resonant orbit to a low prograde orbit around Triton. These transfers span flight times of 18.22$-$72.57 days and a total $\Delta v$ of 0.91$-$4.58 km/s with impulsive maneuvers near the locations of maximum curvature in the rotating frameand at the boundary conditions. In both cases, the motion primitive approach automatically generates transfers that closely resemble their initial guesses. Furthermore, these transfers possess distinct geometries by completing a distinct number of revolutions around various reference points, possessing different shapes, passing through different regions of a system, or traveling in distinct directions. This capability supports automatically generating trajectories that span a complex tradespace. Future work may include leveraging this capability to analyze the broader tradespace with various initial conditions and science orbits, additional mission-derived constraints, specific scientific objectives, and spatial transfers.

## Supplementary Information

Below is the link to the electronic supplementary material.

Supplementary file 1 (pdf 982 KB)

Supplementary file 2 (txt 13 KB)

Supplementary file 3 (txt 42 KB)

Supplementary file 4 (txt 107 KB)

## Data Availability

Data describing the motion primitives for each of the technical approach and two scenarios in the Results section are provided in supplementary data files (Online Resources 2, 3, and 4).

## Code Availability

No additional codes are provided.

## References

[1] National Academies of Sciences, Engineering, and Medicine: NASA 2023 Decadal Survey. Technical report, National Academies Press, Washington, DC (2023)

[2] Vallado, D.A.: Fundamentals of Astrodynamics and Applications, 5th edn. Microcosm Press, New York (2022)

[3] Marley, D. M. L.: Planetary Science Decadal Survey JPL Rapid Mission Architecture Neptune-Triton-KBO Study Final Report. Technical Report, NASA Jet Propulsion Laboratory, Pasadena, CA (2010)

[4] Masters, A., Achilleos, N., Agnor, C.B., Campagnola, S., Charnoz, S., Christophe, B., Coates, A.J., Fletcher, L.N., Jones, G.H., Lamy, L., Marzari, F., Nettelmann, N., Ruiz, J., Ambrosi, R., Andre, N., Bhardwaj, A., Fortney, J.J., Hansen, C.J., Helled, R., Moragas-Klostermeyer, G., Orton, G., Ray, L., Reynaud, S., Sergis, N., Srama, R., Volwerk, M.: Neptune and Triton: essential pieces of the solar system puzzle. Planet. Space Sci. **104**, 108–121 (2014). 10.1016/j.pss.2014.05.008

[5] Melman, J., Orlando, G., Safipour, E., Mooij, E., Noomen, R.: Trajectory optimization for a mission to Neptune and Triton. In: AIAA/AAS Astrodynamics Specialist Conference, Honolulu (2008)

[6] Koon, W.S., Lo, M.W., Marsden, J.E., Ross, S.D.: Dynamical Systems, the Three-Body Problem, and Space Mission Design. (2011). https://www.cds.caltech.edu/~marsden/books/Mission_Design.html

[7] Campagnola, S., Russell, R.P.: Endgame problem part 2: multibody technique and the Tisserand-Poincare graph. J. Guid. Control Dyn. **33**(2), 476–486 (2010). 10.2514/1.44290

[8] Campagnola, S., Skerritt, P., Russell, R.P.: Flybys in the planar, circular, restricted, three-body problem. Celest. Mech. Dyn. Astron. **113**(3), 343–368 (2012). 10.1007/s10569-012-9427-x

[9] Ross, S.D., Scheeres, D.J.: Multiple gravity assists, capture, and escape in the restricted three-body problem. SIAM J. Appl. Dyn. Syst. **6**(3), 576–596 (2007). 10.1137/060663374

[10] Villac, B.F.: Using FLI maps for preliminary spacecraft trajectory design in multi-body environments. Celest. Mech. Dyn. Astron. **102**(1), 29–48 (2008). 10.1007/s10569-008-9158-1

[11] Guzzetti, D., Bosanac, N., Haapala, A., Howell, K.C., Folta, D.C.: Rapid trajectory design in the Earth-Moon ephemeris system via an interactive catalog of periodic and quasi-periodic orbits. Acta Astronaut. **126**, 439–455 (2016). 10.1016/j.actaastro.2016.06.029

[12] Restrepo, R.L., Russell, R.P.: A database of planar axisymmetric periodic orbits for the solar system. Celest. Mech. Dyn. Astron. **130**(7), 49 (2018). 10.1007/s10569-018-9844-6

[13] Tsirogiannis, G.A.: A graph based methodology for mission design. Celest. Mech. Dyn. Astron. **114**(4), 353–363 (2012)

[14] Trumbauer, E.M.: Rapid Onboard Trajectory Design for Autonomous Spacecraft in Multibody Systems. PhD thesis, University of California, Irvine (2015)

[15] Das-Stuart, A., Howell, K.C., Folta, D.C.: Rapid trajectory design in complex environments enabled by reinforcement learning and graph search strategies. Acta Astronaut. **171**, 172–195 (2020). 10.1016/j.actaastro.2019.04.037

[16] Sullivan, C.J.: Low-Thrust Trajectory Design in Multi-body Systems via Multi-Objective Reinforcement Learning. PhD Thesis, University of Colorado at Boulder (2022)

[17] LaFarge, N.B., Miller, D., Howell, K.C., Linares, R.: Autonomous closed-loop guidance using reinforcement learning in a low-thrustmulti-body dynamical environment. Acta Astronaut. **186**, 1–23 (2021). 10.1016/j.actaastro.2021.05.014

[18] Bruchko, K.L., Bosanac, N.: Rapid trajectory design in multi-body systems using sampling-based kinodynamic planning. J. Astronaut. Sci. **72**(4), 33 (2025). 10.1007/s40295-025-00506-6

[19] Wolek, A., Woolsey, C.A.: In: Fossen, T.I., Pettersen, K.Y., Nijmeijer, H. (eds.) Model-Based Path Planning, pp. 183–206. Springer, Cham (2017). 10.1007/978-3-319-55372-6_9

[20] Frazzoli, E., Dahleh, M.A., Feron, E.: Maneuver-based motion planning for nonlinear systems with symmetries. IEEE Trans. Robot. **21**(6), 1077–1091 (2005). 10.1109/TRO.2005.852260

[21] Paranjape, A.A., Meier, K.C., Shi, X., Chung, S.J., Hutchinson, S.: Motion primitives and 3D path planning for fast flight through a forest. Int. J. Robot. Res. **34**(3), 357–377 (2015). 10.1177/0278364914558017

[22] Wang, B., Gong, J., Zhang, R., Chen, H.: Learning to segment and represent motion primitives from driving data for motion planning applications. In: 21st International Conference on Intelligent Transportation Systems, Maui, pp. 1408–1414 (2018). 10.1109/ITSC.2018.8569913

[23] Reng, L., Moeslund, T.B., Granum, E.: Finding motion primitives in human body gestures. In: Gesture in Human-Computer Interaction and Simulation: 6th International Gesture Workshop, Berder Island, pp. 133–144 (2005). 10.1007/11678816_16

[24] Majumdar, A., Tedrake, R.: Funnel libraries for real-time robust feedback motion planning. Int. J. Robot. Res. **36**(8), 947–982 (2017). 10.1177/0278364917712421

[25] Smith, T.R., Bosanac, N.: Motion primitive approach to spacecraft trajectory design in a multi-body system. J. Astronaut. Sci. **70**(5), 34 (2023). 10.1007/s40295-023-00395-7 37706006 10.1007/s40295-023-00395-7 PMC10495503

[26] Smith, T.R., Bosanac, N.: Constructing motion primitive sets to summarize periodic orbit families and hyperbolic invariant manifolds in a multi-body system. Celest. Mech. Dyn. Astron. **134**(1), 7 (2022). 10.1007/s10569-022-10063-x

[27] Gillespie, C., Miceli, G.E., Bosanac, N.: Summarizing natural and controlled motion in cislunar space with behavioral motion primitives. In: AAS/AIAA Space Flight Mechanics Meeting, Kauai (2025)

[28] NASA Navigation and Ancillary Information Facility (NAIF): NASA Navigation and Ancillary Information Facility (NAIF) Generic Kernels. https://naif.jpl.nasa.gov/pub/naif/generic_kernels/

[29] Szebehely, V.: Theory of Orbits: The Restricted Problem of Three Bodies. Academic Press, London (1967)

[30] National Aeronautics and Space Administration (NASA): Planetary Fact Sheets: Neptune and Neptunian Satellites. https://nssdc.gsfc.nasa.gov/planetary/planetfact.html

[31] Acton, C.H.: Ancillary data services of NASA’s navigation and ancillary information facility. Planet. Space Sci. **44**(1), 65–70 (1996). 10.1016/0032-0633(95)00107-7

[32] Acton, C., Bachman, N., Semenov, B., Wright, E.: A look towards the future in the handling of space science mission geometry. Planet. Space Sci. **150**, 9–12 (2018). 10.1016/j.pss.2017.02.013

[33] Park, R.S., Folkner, W.M., Williams, J.G., Boggs, D.H.: The JPL planetary and lunar ephemerides DE440 and DE441. Astron. J. **161**(3), 105 (2021). 10.3847/1538-3881/abd414

[34] Charlot, P., Jacobs, C.S., Gordon, D., Lambert, S., de Witt, A., Böhm, J., Fey, A.L., Heinkelmann, R., Skurikhina, E., Titov, O., Arias, E.F., Bolotin, S., Bourda, G., Ma, C., Malkin, Z., Nothnagel, A., Mayer, D., MacMillan, D.S., Nilsson, T., Gaume, R.: The third realization of the international celestial reference frame by very long baseline interferometry. Astron. Astrophys. **644**, 159 (2020). 10.1051/0004-6361/202038368

[35] Murray, C.D., Dermott, S.F.: Solar System Dynamics. Cambridge University Press, Cambridge (1999)

[36] Vaquero Escribano, T.M.: Spacecraft Transfer Trajectory Design Exploiting Resonant Orbits in Multi-body Environments. PhD Thesis, Purdue University, West Lafayette (2013)

[37] Henon, M.: Numerical exploration of the restricted problem, V. Astron. Astrophys. **1**, 223–238 (1969)

[38] Parker, J.S., Anderson, R.L.: Low-Energy Lunar Trajectory Design. Wiley, Hoboken (2014)

[39] Patrikalakis, N.M., Maekawa, T., Cho, W.: Shape Interrogation for Computer Aided Design and Manufacturing. Springer, Berlin, Heidelberg. 10.1007/978-3-642-04074-0

[40] Bosanac, N.: Curvature extrema along trajectories in the circular restricted three-body problem. In: AAS/AIAA Astrodynamics Specialists Conference (2024)

[41] Han, J., Kamber, M., Pei, J.: Data Mining: Concepts and Techniques. Morgan Kaufmann Publishers, Waltham (2012)

[42] Ester, M., Kriegel, H.-P., Sander, J., Xu, X.: A density-based algorithm for discovering clusters in large spatial databases with noise. In: Proceedings of the Second International Conference on Knowledge Discovery and Data Mining (KDD’96), pp. 226–231. AAAI Press, Portland (1996)

[43] Campello, R., Moulavi, D., Sander, J.: Density-based clustering based on hierarchical density estimates. In: Pei. J, et al. (eds.) Advances in Knowledge Discovery and Data Mining. PAKDD 2013. Lecture Notes in Computer Science, vol. 7819, pp. 160–172. Springer, Berlin, Heidelberg (2013). 10.1007/978-3-642-37456-2_14

[44] Bosanac, N.: Data-mining approach to Poincaré maps in multi-body trajectory design. J. Guid. Control Dyn. **43**(6), 1190–1200 (2020). 10.2514/1.G004857 32831465 10.2514/1.G004857 PMC7430177

[45] Bosanac, N.: Data-driven summary of motion in an ephemeris model of cislunar space. In: AAS/AIAA Space Flight Mechanics Meeting, Lihue (2025)

[46] Malzer, C., Baum, M.: A hybrid approach to hierarchical density-based cluster selection. In: 2020 IEEE International Conference on Multisensor Fusion and Integration for Intelligent Systems (MFI), pp. 223–228 (2020). 10.1109/MFI49285.2020.9235263

[47] McInnes, L., Healy, J., Astels, S.: hdbscan: hierarchical density based clustering. J. Open Source Softw. **2**(11), 205 (2017). 10.21105/joss.00205

[48] Bosanac, N., Joyner, M.: Data-driven summary of continuous thrust trajectories in a low-fidelity model of cislunar space. In: AAS/AIAA Astrodynamics Specialist Conference, Broomfield (2024)

[49] Zheng, Y., Zhou, X.: Computing with Spatial Trajectories. Springer, New York (2011). 10.1007/978-1-4614-1629-6

[50] MATLAB: Version R2022a. The MathWorks Inc., Natick (2022)

[51] Hart, P.E., Nilsson, N.J., Raphael, B.: A formal basis for the heuristic determination of minimum cost paths. IEEE Trans. Syst. Sci. Cybern. **4**(2), 100–107 (1968). 10.1109/TSSC.1968.300136

[52] Yen, J.: An algorithm for finding shortest routes from all source nodes to a given destination in general networks. Q. Appl. Math. **27**, 526–530 (1970). 10.1090/qam/253822

[53] Ortega-Arranz, H., Gonzalez-Escribano, A., Llanos, D.R.: The Shortest-Path Problem: Analysis and Comparison of Methods. Springer, Cham (2022). 10.1007/978-3-031-02574-7

[54] LaValle, S.M.: Planning Algorithms. Cambridge University Press, Cambridge (2006)

[55] Conway, B.A.: Spacecraft Trajectory Optimization. Cambridge University Press, New York (2010)

[56] Grebow, D.J., Pavlak, T.A.: MCOLL: Monte collocation trajectory design tool. In: AAS/AIAA Astrodynamics Specialist Conference, Stevenson (2017). https://hdl.handle.net/2014/46415

[57] Smith, T.R.: Using Motion Primitives to Rapidly Design Trajectories in Multi-body Systems. PhD Dissertation, University of Colorado Boulder, Boulder (2023)

[58] Williams, P.: Hermite-Legendre-Gauss-Lobatto direct transcription in trajectory optimization. J. Guid. Control Dyn. **32**(4), 1392–1395 (2009). 10.2514/1.42731

[59] De Boor, C.: Good approximation by splines with variable knots. II. In: Proceedings of the Conference on the Numerical Solution of Differential Equations, Dundee (1973)

[60] Russell, R.D., Christiansen, J.: Adaptive mesh selection strategies for solving boundary value problems. SIAM J. Numer. Anal. **15**(1), 59–80 (1978)

[61] Parrish, N.L., Scheeres, D.J.: Low-thrust trajectory optimization with simplified SQP algorithm. In: AAS/AIAA Astrodynamics Specialist Conference (2017). https://ntrs.nasa.gov/citations/20170007868

[62] Miceli, G.E., Bosanac, N., Stuart, J.R., Alibay, F.: Motion primitive approach to spacecraft trajectory design in the neptune–triton system. In: AIAA SciTech Forum, Orlando (2024)

