# Supplementary material for: Generating Planar Trajectories for Neptunian System Exploration Using Motion Primitives
Source: J Astronaut Sci. 2026 Feb 10;73(1):11. doi: 10.1007/s40295-025-00545-z (PMC12891113; doi:10.1007/s40295-025-00545-z)
Supplement: Supplementary file 1 — (pdf 982 KB) [file 40295_2025_545_MOESM1_ESM.pdf]

The following tables and flowcharts summarize the governing parameters and procedures described in the following manuscript: Miceli, G.E., and Bosanac, N, 'Generating Planar Trajectories for Neptunian System Exploration Using Motion Primitives', The Journal of the Astronautical Sciences, 2025. This procedure was developed by Dr. Giuliana Miceli (giuliana.miceli@colorado.edu) and Dr. Natasha Bosanac (natasha.bosanac@colorado.edu) at the University of Colorado Boulder.

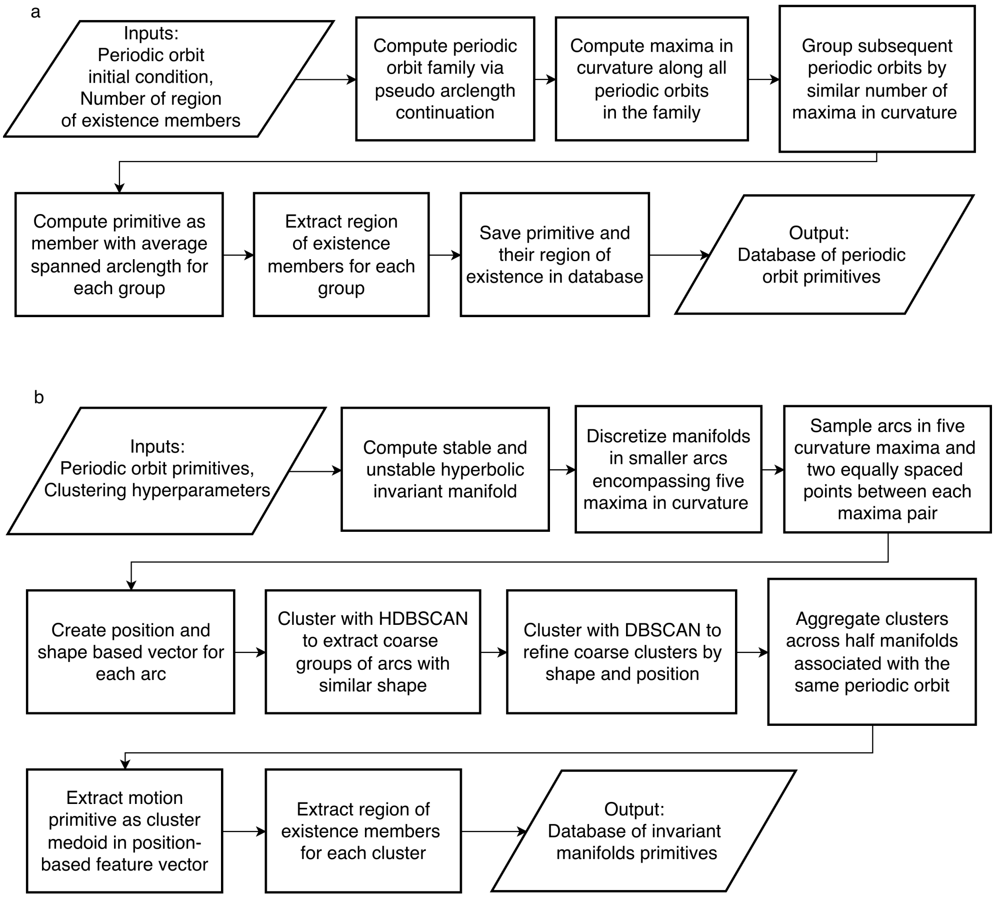

Fig. 1 Flowcharts of process used in Step 1 to generate motion primitives from a) periodic orbits and b) hyperbolic stable and unstable invariant manifolds

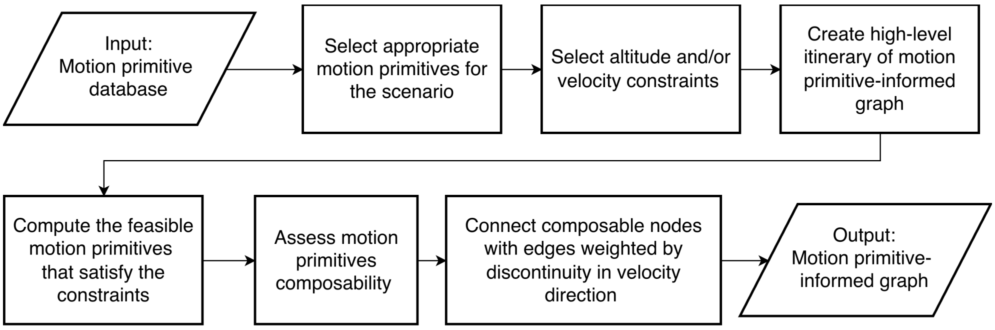

Fig. 2 Flowcharts of the process used in step 2 to generate motion primitive-informed graph

Supplementary material for: Miceli, G.E., and Bosanac, N, 'Generating Planar Trajectories for Neptunian System Exploration Using Motion Primitives', The Journal of the Astronautical Sciences, 2025.

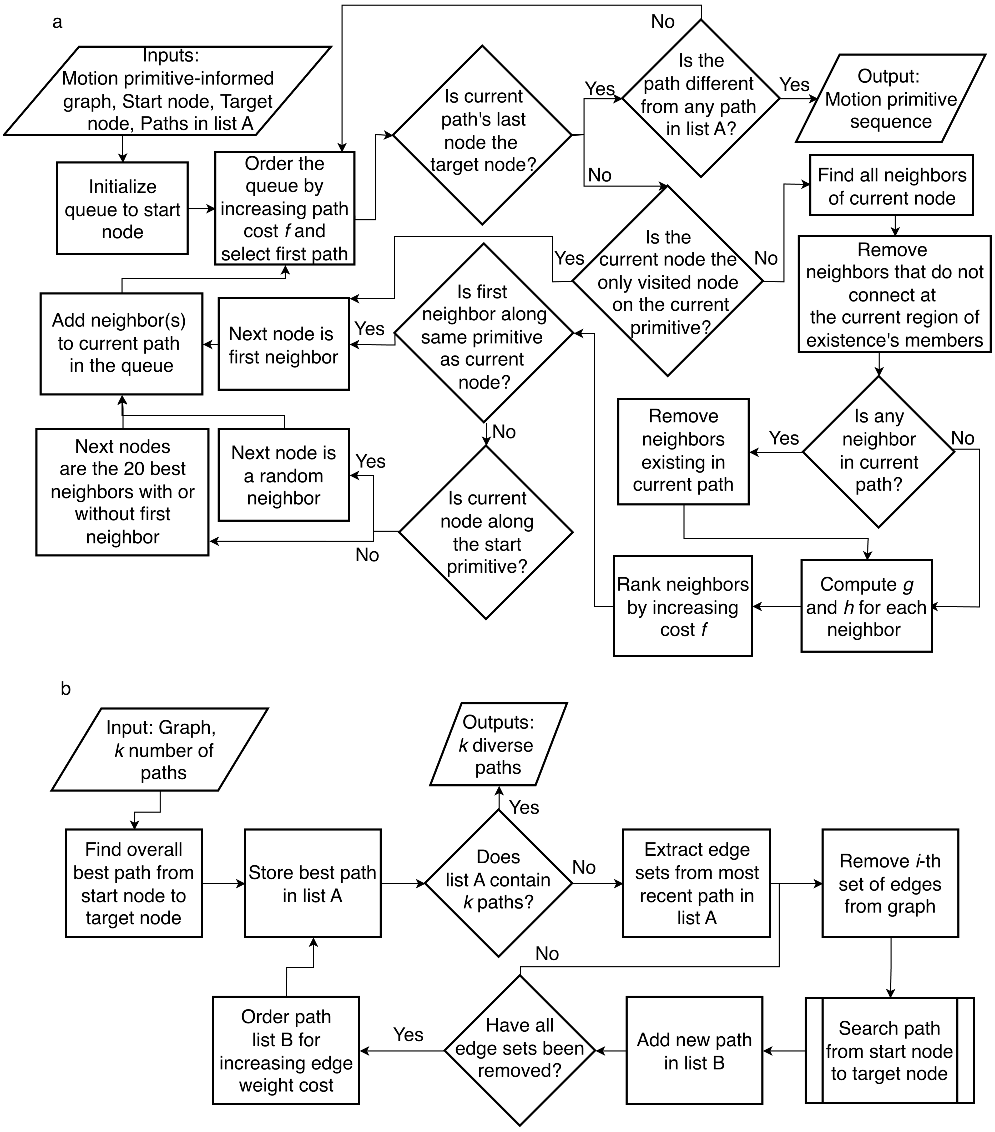

Fig. 3 Flowcharts of search algorithms used in Step 3 to generate a) one sequence of motion primitives and b) a diverse set of k optimal motion primitive sequences.

Supplementary material for: Miceli, G.E., and Bosanac, N, 'Generating Planar Trajectories for Neptunian System Exploration Using Motion Primitives', The Journal of the Astronautical Sciences, 2025.

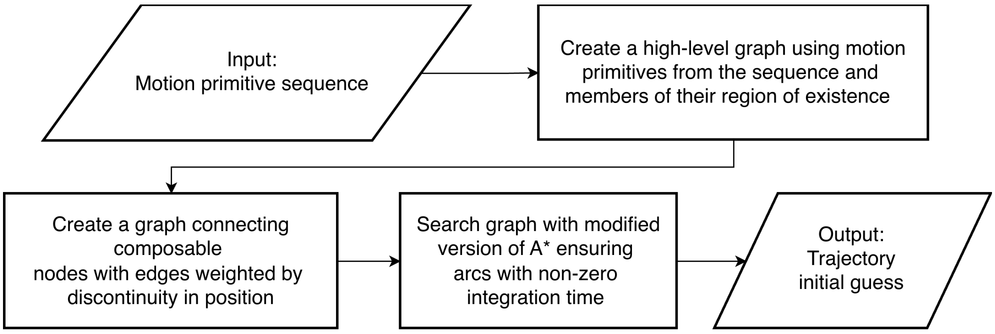

Fig. 4 Flowcharts of the process used in Step 4 to generate a trajectory initial guess from a motion primitive sequence.

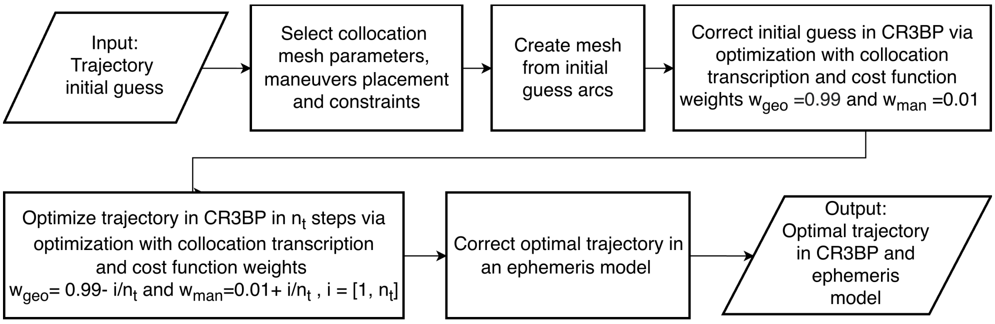

Fig. 5 Flowcharts of the process used in Step 5 to generate an optimal trajectory in an ephemeris model from a trajectory initial guess

**Governing parameter**	**Influence**	**Value**
Arc definition along stable/unstable manifolds	Supplies consistent definition for sampling arcs from a longer trajectory that lies along a stable/unstable manifold	Arcs encompass 5 maxima in curvature
Sample arcs generated from stable/unstable manifolds	Sampled states are used to generate feature vectors for clustering and construct nodes in the motion primitive-informed graph	$n_L = 13$ samples: 5 at maxima in curvature and 2 equally spaced in arc length between consecutive maxima in curvature
Number of trajectories sampled to approximate region of existence	Coarsely approximates the region of the phase space where arcs of similar geometries exist and influences the initial guess refinement by providing more options for arcs to sequentially compose	50 members from a cluster of arcs along a stable or unstable manifold and 20 members for periodic orbit groups, equally spaced in the position vector space from the primitive
Parameters governing HDBSCAN	Govern the initial coarsely clustered groups of arcs generated from stable/unstable manifolds. Smaller values prioritize discovering localized variations between trajectories.	$n_{minsize} = 5$, $n_{core} = 4$, $\epsilon_{merge} = 2\sqrt{13}\ \sin(5°)$
Parameters governing DBSCAN	Govern the refined clusters of arcs generated from stable/unstable manifolds. Smaller values prioritize discovering localized variations between trajectories.	$n_{pts} = n_{min,core} = 4$ and $\epsilon = (n_{pts}+1)\max(e, \epsilon_{threshold})$ where $\epsilon_{threshold}$ is $2\sin(2.5°)$ and 10^{-3} in the shape-based and position-based feature vector spaces, respectively, and e is the $n_{minsize}$-largest distance between each member and its nearest neighbor.
Motion primitive-informed graph node definition	Governs the discrete representation of the primitives in the state space.	Nodes are defined as states sampled at maxima in curvature in the rotating frame and at 2 (manifold arcs) or 5 (periodic orbits) nodes equally spaced in arclength between consecutive curvature maxima.
Parameters for sequential composability	Influence the identification of composable nodes based on position and velocity discontinuity.	For position composability, the two states of the nodes must have overlapping neighborhoods in position, defined with an average radius equal to the distance to the nearest neighbor, multiplied by $c = 1.5$. For velocity discontinuity in Equation 8 $\theta_{max} = 30°$.
Number of neighbors r to explore in A*	Influences how many neighboring nodes are explored at each iteration before arriving at the target node.	$r = 1$ if the next node is along the same primitive as the current node or if the current node is the first along a primitive or is along the primitive representing the transfer's initial conditions, otherwise $r = 20$.
Collocation polynomial order	Affects the accuracy of the correction	7th-order polynomial.
Objective function scalar weights	Influence the properties of the final trajectory	Cost function as in Equation 19, with weights modified from $[w_{geo}, w_{man}] = [0.99, 0.01]$ for correcting the initial guess to $[w_{geo}, w_{man}] = [0.01, 0.99]$ for optimization.
Correction tolerances	Influence the numerical accuracy of the final continuous trajectory	In the CR3BP $tol = 10^{-12}$ and in the ephemeris model $tol = 10^{-9}$
Optimality tolerances	Influences the numerical accuracy of the solution obtained during the optimization process	10^{-3}

Table 1 Table of governing parameters for the motion primitive trajectory design technical approach
